# Genome-Wide Identification and Expression Analyses of the bZIP Transcription Factor Genes in moso bamboo (*Phyllostachys edulis*)

**DOI:** 10.3390/ijms20092203

**Published:** 2019-05-05

**Authors:** Feng Pan, Min Wu, Wenfang Hu, Rui Liu, Hanwei Yan, Yan Xiang

**Affiliations:** 1 Laboratory of Modern Biotechnology, School of Forestry and Landscape Architecture, Anhui Agricultural University, Hefei 230036, China; fengpan1231@163.com (F.P.); wenfanghu0@163.com (W.H.); liurui940510@163.com (R.L.); hwyanahau@163.com (H.Y.); 2 Key Laboratory of Crop Biology of Anhui Province, School of Life Sciences, Anhui Agricultural University, Hefei 230036, China; minwu111@163.com

**Keywords:** moso bamboo, bZIP genes, transcription factor, expression patterns

## Abstract

The basic leucine zipper (bZIP) transcription factor (TF) family is one of the largest gene families, and play crucial roles in many processes, including stress responses, hormone effects. The TF family also participates in plant growth and development. However, limited information is available for these genes in moso bamboo (*Phyllostachys edulis*), one of the most important non-timber forest products in the world. In the present study, 154 putative *PhebZIP* genes were identified in the moso bamboo genome. The phylogenetic analyses indicate that the *PhebZIP* gene proteins classify into 9 subfamilies and the gene structures and conserved motifs that analyses identified among all *PhebZIP* proteins suggested a high group-specificity. Microsynteny and evolutionary patterns analyses of the non-synonymous (Ka) and synonymous (Ks) substitution rates and their ratios indicated that paralogous pairs of *PhebZIP* genes in moso bamboo underwent a large-scale genome duplication event that occurred 7–15 million years ago (MYA). According to promoter sequence analysis, we further selected 18 genes which contain the higher number of *cis*-regulatory elements for expression analysis. The result showed that these genes are extensively involved in GA-, ABA- and MeJA-responses, with possibly different mechanisms. The tissue-specific expression profiles of *PhebZIP* genes in five plant tissues/organs/developmental stages suggested that these genes are involved in moso bamboo organ development, especially seed development. Subcellular localization and transactivation activity analysis showed that *PhebZIP47* and *PhebZIP126* were localized in the nucleus and *PhebZIP47* with no transcriptional activation in yeast. Our research provides a comprehensive understanding of *PhebZIP* genes and may aid in the selection of appropriate candidate genes for further cloning and functional analysis in moso bamboo growth and development, and improve their resistance to stress during their life.

## 1. Introduction

Transcription factor (TF) proteins play crucial roles in activating gene expression by binding to the promoter regions of target genes. The basic leucine zipper (bZIP) domain encoding genes, generally associated with stress resistance and control many crucial biological processes in plants, form one of the largest TF families in eukaryotes, and are named for a conserved bZIP domain containing a basic region and a leucine zipper sequence of 60–80 amino acid residues [1]. The basic region has a conserved N-x_7_-R/K-x_9_ motif, which is responsible for DNA binding and nuclear localization, while the leucine zipper region is composed of several heptad repeats of leucine residues or other bulky hydrophobic amino acid, such as isoleucine, valine, phenylalanine, or methionine [2].

To date, the bZIP TF genes have been predicted or identified using genome-wide analyses in multiple plants. Seventy-five bZIP TF genes have been found in *Arabidopsis thaliana* [3]. In addition, the genes have also been characterized in several grass family (*Gramineae*) species, including 89 bZIP TF genes in *Oryza sativa* [4], 92 in *Sorghum bicolor* [5], 125 in *Zea mays* [6], 96 in *Brachypodium distachyon* [7], and 187 in *Triticum aestivum* [8]. In addition, increasing evidence also shows that bZIP TFs play essential roles in regulating the integration and development of many tissues and organs, including vascular development [9], floral induction and development [10,11,12,13,14,15,16,17], seed maturation and germination [18,19], and embryogenesis [20,21]. Other studies also have found that bZIP TFs to be crucially involved in various plant hormone and stress responses, including abscisic acid (ABA) [22], gibberellin (GA) [23], ethylene [24], pathogen infection [25], drought [26], cold [27], heat [28], and high salinity stress [29]. For example, the model plant *Arabidopsis thaliana* bZIP TF genes (*AtbZIPs*) *AtbZIP36*, *AtbZIP37*, and *AtbZIP38* are up-regulated in response to drought, salt stress, and following ABA treatment [30]. In more recent studies, Hossain et al (2010a,b) [31,32] found that two *Oryza sativa* bZIP TF genes (*OsbZIPs*), *OsbZIP12* and *OsbZIP46*, are also strongly up-regulated by the stresses mentioned above, as well as by phytohormone treatment. Overall, the bZIP TFs play a vital role in various processes, protecting plants against a variety of stresses, and are also involved in responses to various plant hormone signals [33].

Moso bamboo (*Phyllostachys edulis*) belongs to the Poaceae family, represents the only major lineage of grasses that is native to forests, is one of the most important non-timber forest products and has great ecological, cultural, and economic value in the world [34]. It is used as an important source of timber, food, and paper material in daily life [34]. Extreme climate and unfavorable growth conditions such as excess salinity, soil depletion, drought, cold and heat stress, and wounding largely limit the growth of moso bamboo. The moso bamboo has evolved various adaptive mechanisms to deal with varied, adverse environmental factors, and survive those stresses. To our knowledge, however, very limited information is available regarding the bZIP TF gene family in moso bamboo, its multiple roles in stress and hormone effects not yet studied, until now. We want to preliminarily explore the function of bZIP TF gene in the stress and hormone response, and whether these genes play an important role in regulating growth and development as well as stress resistance in moso bamboo during their life.

We identified 154 putative bZIP TF genes (*PhebZIPs*) from the *P. edulis* genome, and investigated genomic structure and phylogenetic relationships with counterparts from the other Poaceae species *O. sativa* and *B. distachyon.* We systematically analyzed conserved motifs, microsynteny and evolutionary patterns of the encoded *PhebZIP* proteins, and located *cis*-regulatory-elements in the genomic sequences. According to the number of the *cis*-regulatory elements, 18 genes were selected to examine the expression of *PhebZIP* genes in response to three different phytohormones, as well as in different tissues/organs/developmental stages. For deeper study, we cloned two genes from 18 genes and analyzed their subcellular localization. In addition, the *PhebZIP47* gene of these two genes was highly expressed in all hormone treatments and tissues was selected for transactivation activity analysis. In general, we provide a basis for the screening of appropriate candidate *PhebZIP* genes for further cloning to know the molecular mechanisms in hormone response and improve the stress resistance of moso bamboo.

## 2. Results

### 2.1. Identification of bZIP Genes in moso bamboo

The candidate *PhebZIPs* were named based on chromosomal locations, *PhebZIP1* to *PhebZIP154*, from top to bottom, in the present study. The proteins encoded by the 154 predicted full-length *PhebZIP* genes range from 80 (*PhebZIP15*) to 839 (*PhebZIP134*) amino acids (aa), and from 8679.74 to 91904.76 Da in relative molecular weight. The isoelectric points (PIs) of the *PhebZIP* proteins are predicted to range from 4.7 (*PhebZIP28*) to 11.73 (*PhebZIP141*). Detailed information for the examined bZIP proteins is shown in Table S1.

### 2.2. Phylogenetic Analysis of bZIP Proteins in the Three Different Plant Species

**Figure 1:**
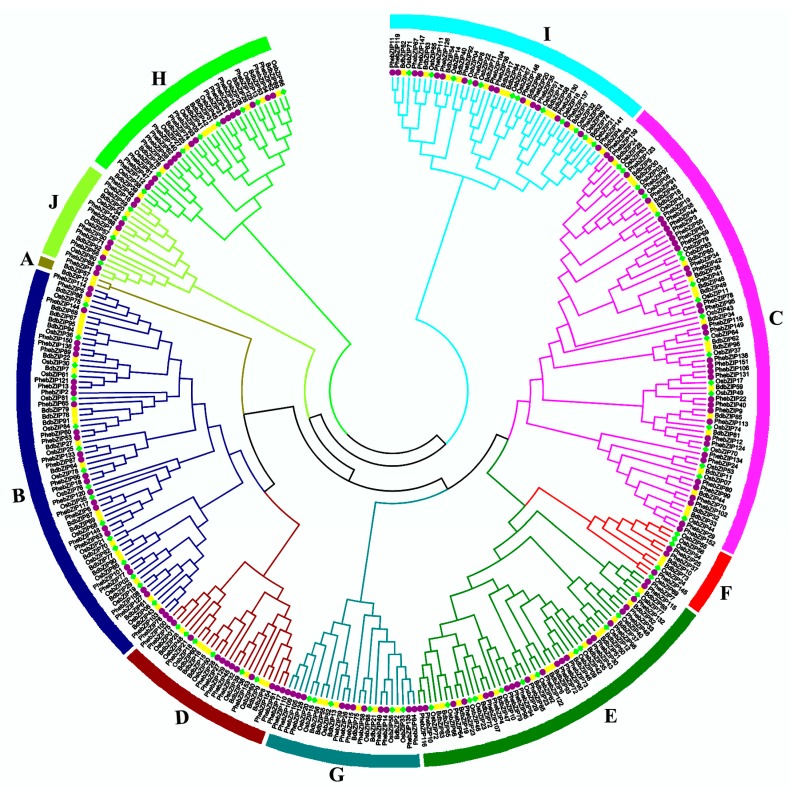
Phylogenetic tree and distribution of bZIP protein from three plant species: rice, *Brachypodium distachyon*, and moso bamboo. The phylogenetic tree was constructed using the neighbor-joining method as implemented in MEGA6.0 from a bZIP protein sequence alignment. Bootstrap values from 1000 replicates are displayed at each node. The proteins on the tree can be divided into ten clades and the different clades are indicated by different colors.

A comprehensive phylogenetic tree containing 339 bZIP gene protein sequences from three monocot species (96 from *B. distachyon*, 89 from the *O. sativa*, and 154 from *P. edulis*) was estimated (Figure 1). The *OsbZIP* and *BdbZIP* sequences were obtained, and we generated a phylogenetic tree using the Neighbor-joining (NJ) algorithm in MEGA software (MEGA6.0) with the *PhebZIP* sequences. Detailed bZIP TF gene information from rice and *B. distachyon* is given in Table S2. According to the comprehensive phylogenetic tree, the result showed that the predicted *PhebZIP* gene family cluster into 9 subfamilies, named groups B–J. The three tested monocot species had representatives in most all subfamilies, group A only contains *B. distachyon* sequence.

### 2.3. PhebZIP Gene Structure, Conserved Protein Motifs

**Figure 2:**
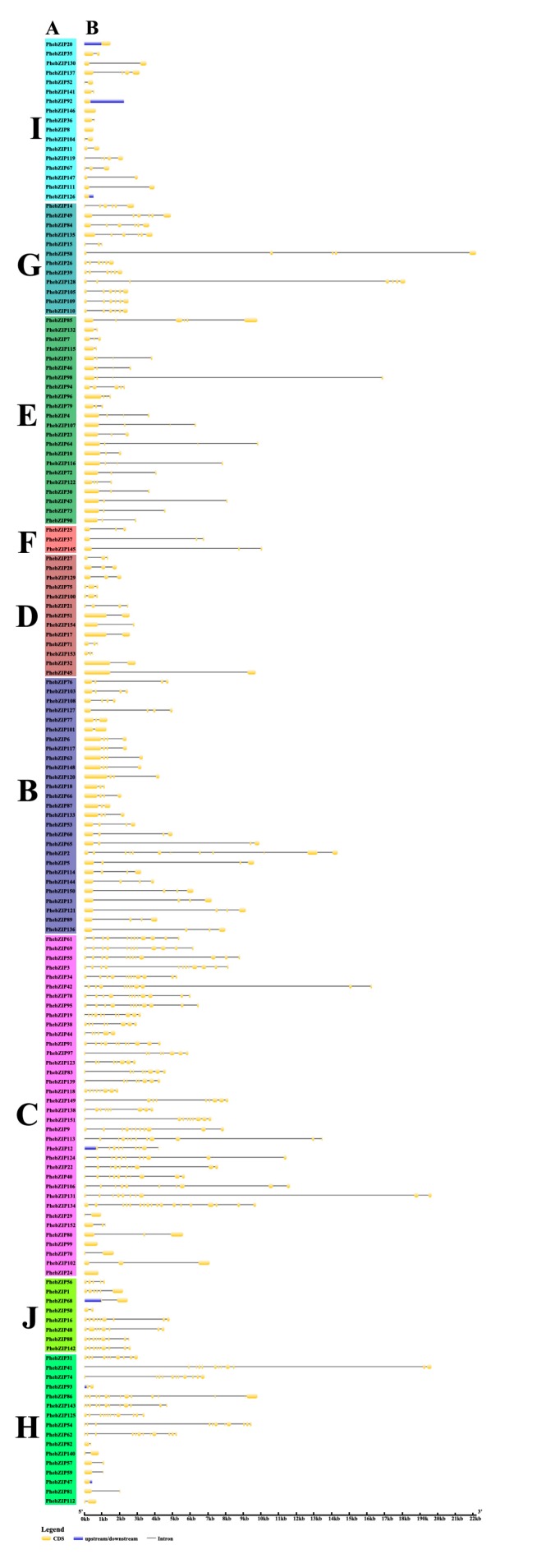
Unrooted tree of the *PhebZIP* gene family and exon/intron organization of moso bamboo (*Phyllostachys edulis*) bZIP proteins. (**A**) The numbers above or below branches on the phylogenetic tree indicate node bootstrap values are based on 1000 replicates. The unrooted tree was constructed with MEGA v. 6.0 software by the Neighbor-Joining (NJ) method with 1000 bootstrap replicates. The middle set of numerals on the tree represent eight *PhebZIP* groups. (**B**) Yellow rectangles represent exons, grey lines represent introns, and blue boxes represent untranslated regions (UTRs).

The exon-intron structure of the 154 *PhebZIP* genes was examined to obtain insight into the family’s structural evolution (Figure 2). The results showed that the number of introns of *PhebZIP* genes varied from 0 to 17. Interestingly, no intron was detected in *PhebZIP* genes of subfamily I and H. In summary, most of *PhebZIPs* belonging to the same groups showed similar exon-intron structures, which indicates their close evolutionary relationship and the classification of subfamilies.

**Figure 3:**
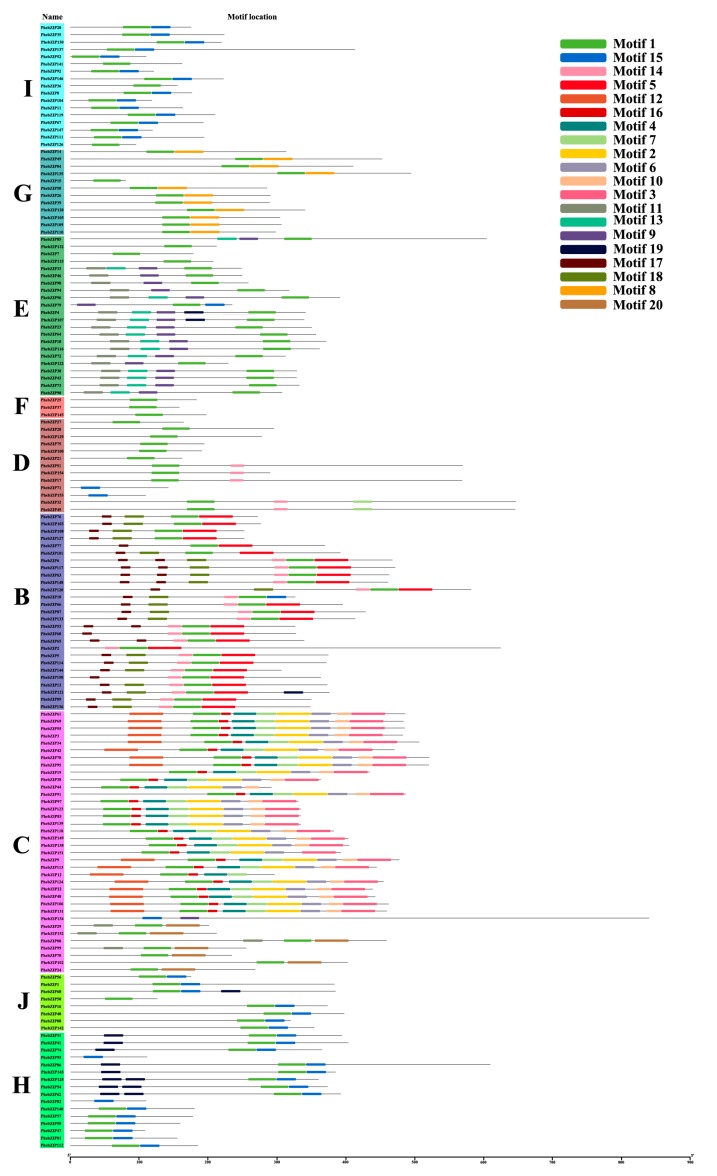
Schematic representation of 20 conserved motifs in *PhebZIP* proteins. Motifs of the *PhebZIP* proteins were identified using the MEME online tool. Each motif is indicated by different colored blocks, with respective numbers in the center of the motifs. The number in boxes (1–20) represents motif 1–motif 20. The position and length of each colored box represents the actual motif size.

The MEME Web server was used to investigate the diversity of motifs in the translations from our candidate *PhebZIP* genes. MEME motifs were captured and designated motif 1 to motif 20 (Figure 3). Notably, almost all of the *PhebZIPs* translations contain motif 1 and subfamily F only contains motif 1. Furthermore, motif 17 is only found in subfamily B and motif 8 also presents exclusively in subfamily G, indicating that these motifs might have a special function. In general, many of the *PhebZIPs* encoded proteins of the same subfamily contain very similar motif compositions and they might have had similar functions.

### 2.4. Chromosomal Location, Microsynteny and Evolutionary Patterns of PhebZIP Genes

**Figure 4:**
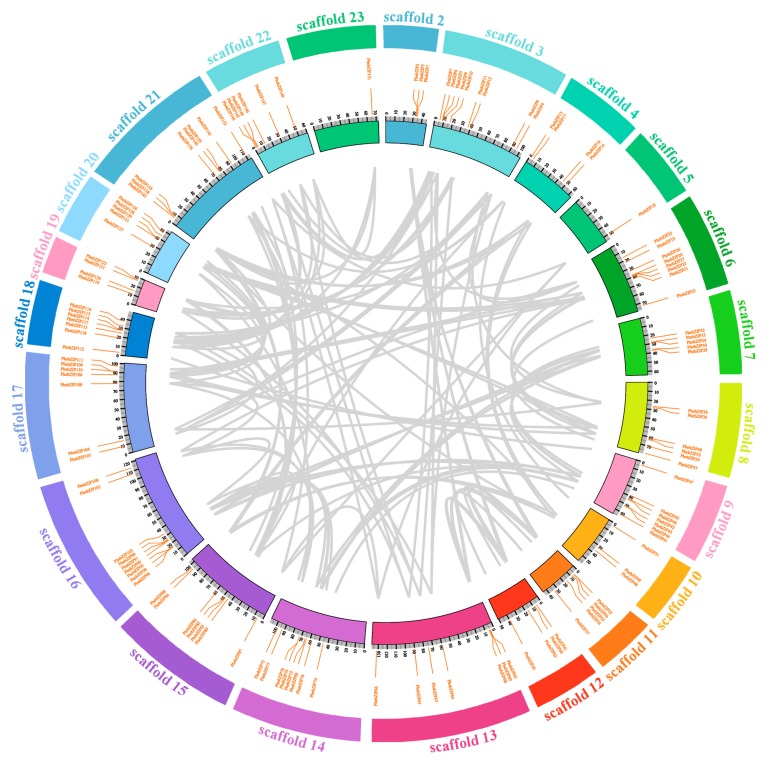
The duplication events of bZIP genes within moso bamboo. The whole chromosomes are shown in a circle. Black lines represent the duplicated gene relationships between bZIP regions.

**Figure 5:**
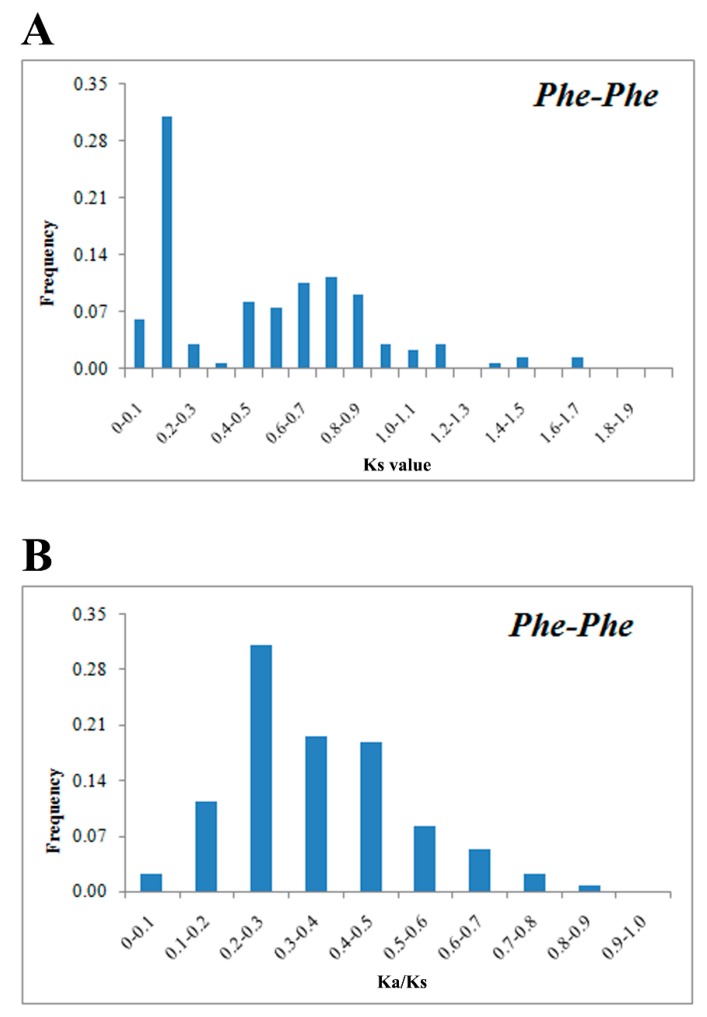
Ks and Ka/Ks value distribution of the bZIP genes in the genomes of moso bamboo paralogous gene-pairs (*Pe*-*Pe*), viewed through the frequency distribution of relative Ks and Ka/Ks modes. Distribution of Ks and Ka/Ks values were obtained from paralogous gene-pairs (*Pe*-*Pe*) in the moso bamboo genome (**A**,**B**).

Gene duplication events play a vital role in the evolution of genes and can lead to the appearance of orthologous and paralogous pairs. According to the chromosomal location map (Figure S1), 154 bZIP genes of moso bamboo were distributed irregularly across 23 chromosomes. To further investigate the evolution of the *PhebZIP* gene family, using microsynteny analysis, we investigated the genome duplication events within moso bamboo (Figure 4). A total of 132 collinear pairs were identified in moso bamboo (Table S3). To estimate evolutionary rates and determine the relative divergence of moso bamboo, the Ks values and Ka/Ks ratios were estimated for the paralogous (Pe-Pe) gene pairs (Figure 5). The frequency distribution of relative Ks values peaked at 0.1–0.2 (Figure 5A), suggesting that a large-scale genome duplication event occurred around 7–15 MYA creating the moso bamboo bZIP TF gene paralogous pairs. A Ka/Ks value of < 1 indicates that a gene underwent negative or purifying selection, while Ka/Ks =1 and > 1 indicate neutral selection, and positive selection, respectively. All the bZIP TF gene paralogue Ka/Ks ratios in moso bamboo are < 1 (Figure 5B), indicating that the *PhebZIP* family paralogous principally underwent purifying selection.

### 2.5. Analysis of PhebZIP cis-Regulatory Elements

**Figure 6:**
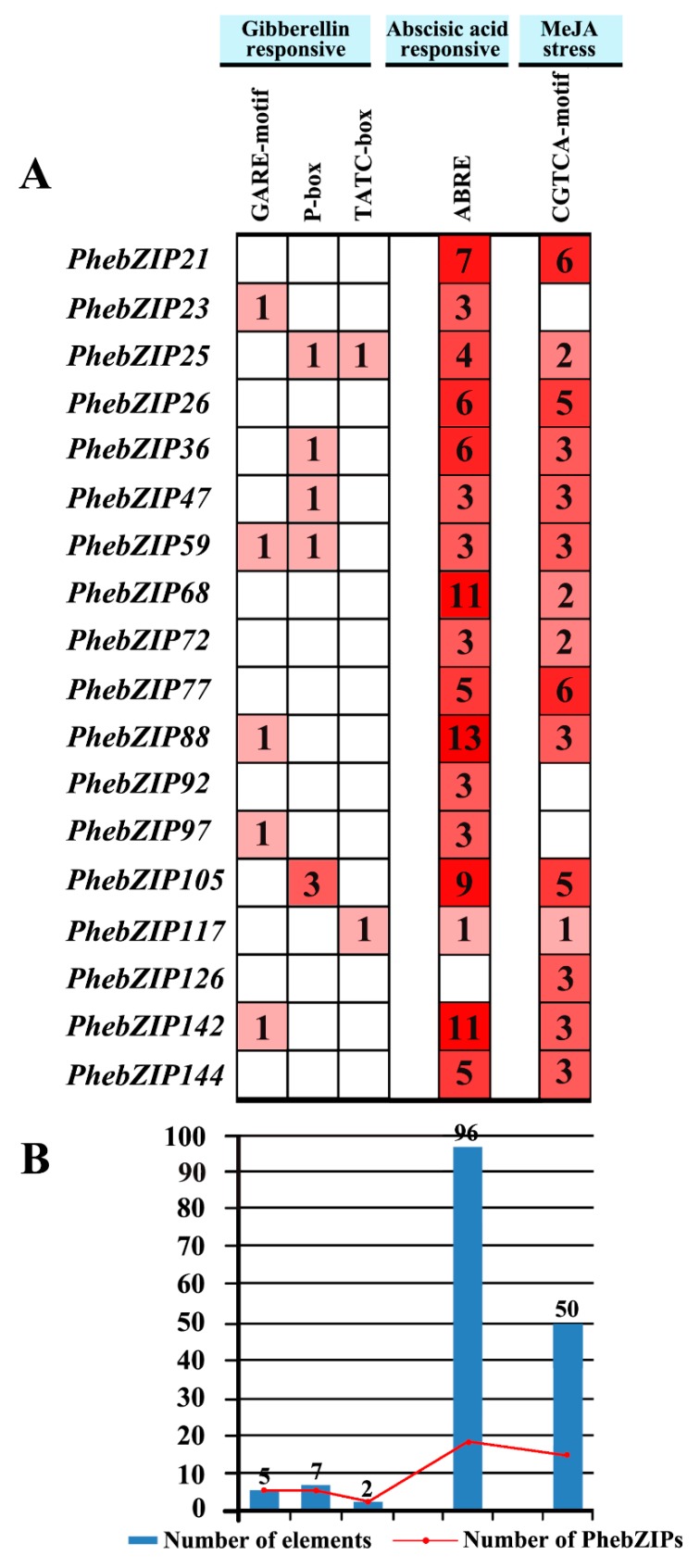
Analysis of *Cis*-acting elements in the promoter regions of *PhebZIP* genes. (**A**) The number of each *cis*-acting element in the promoter regions (2 kb upstream of the translation start site) of *PhebZIP* genes. (**B**) Statistics for the total number of *PhebZIP* genes, including the corresponding *cis*-acting elements (red dots) and the total number of *cis*-acting elements in the *PhebZIP* gene family (blue boxes). Based on functional annotations, the *cis*-acting elements classify into five major groups: gibberellin, abscisic acid, and MeJA.

Gene function and regulation are largely determined by *cis*-regulatory elements [35]. Three types of *cis*-acting elements involved in gibberellin, abscisic acid response and MeJA were identified in *PhebZIP* gene regulatory regions in our study (Table S4). The *cis*-regulatory element analysis shows that the 18 selected *PhebZIP* genes contain many elements associated with GA, ABA and MeJA treatments (Figure 6). Regarding hormone-related *cis*-acting elements, gibberellin responsive elements (GARE-motif, P-box, and TATC-box), abscisic acid responsive elements (ABRE), and MeJA stress responsive elements (CGTCA-motif) are found in the promoters of 10, 17, and 15 *PhebZIP* genes, respectively [36,37,38]. Notably, *PhebZIP88* possesses 13 ABRE elements, *PhebZIP68* and *PhebZIP142* have 11 ABRE elements, respectively. There are 14, 96 and 50 elements that were associated with GA, ABA, and MeJA, respectively. Since these genes had the higher number of *cis*-regulatory elements related to GA-, ABA- and MeJA-responses; were further selected for qRT-PCR analysis.

### 2.6. PhebZIP Gene qRT-PCR Analysis

The *cis*-regulatory elements analysis indicates that the 18 selected *PhebZIP* genes contain a large number of elements which are related to ABA-, GA- and MeJA-responses. Therefore, these results led us to wonder whether these selected genes were up-regulated in these hormone treatments and in different tissues/organs/developmental stages by qRT-PCR analysis.

**Figure 7:**
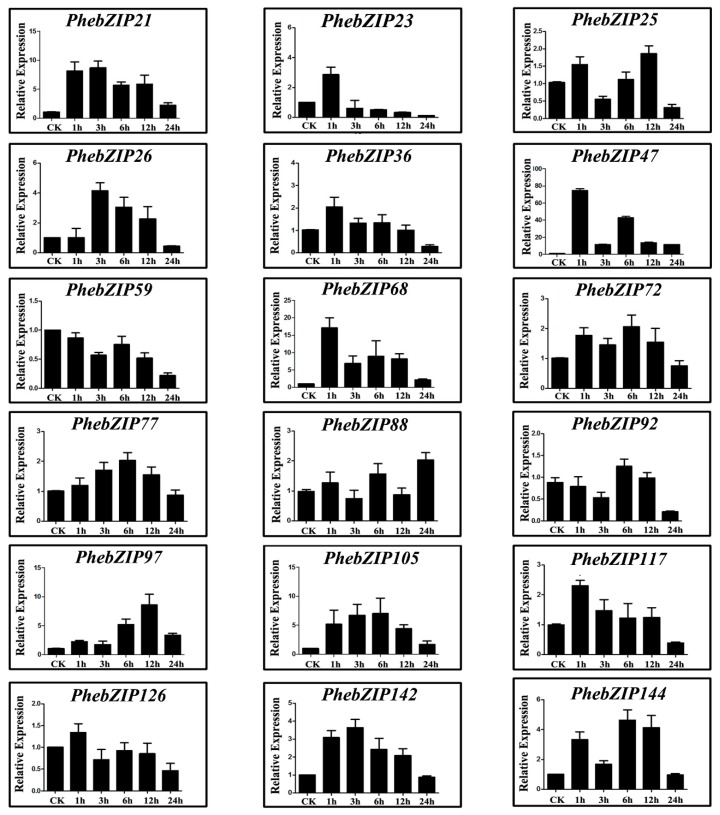
qRT-PCR expression analysis of selected *PhebZIP* genes following methyl jasmonate (MeJA) treatment. Relative expression levels of bZIP genes were examined by qRT-PCR and normalized with respect to the reference gene *TIP41* under drought stress treatment. Bars represent standard deviations (SD) of three biological replicates. Y-axes indicate the scale of the relative expression levels. X-axes show time courses of MeJA stress treatments for each gene.

The MeJA application can stimulate the expression of defense plant genes. Under the treatment, the expression of *PhebZIP21*, −*26*, −*47*, −*68*, −*97*, −*105* and −*144* was up-regulated, while *PhebZIP59* was down-regulated, compared with 0 h (Figure 7). Furthermore, the expression of *PhebZIP47* and *PhebZIP68* peaked at 1 h; *PhebZIP21,* and *PhebZIP26* peaked at 3 h; and *PhebZIP105* and *PhebZIP144* peaked at 6 h. The peak expression of *PhebZIP97* was observed at 12 h.

**Figure 8:**
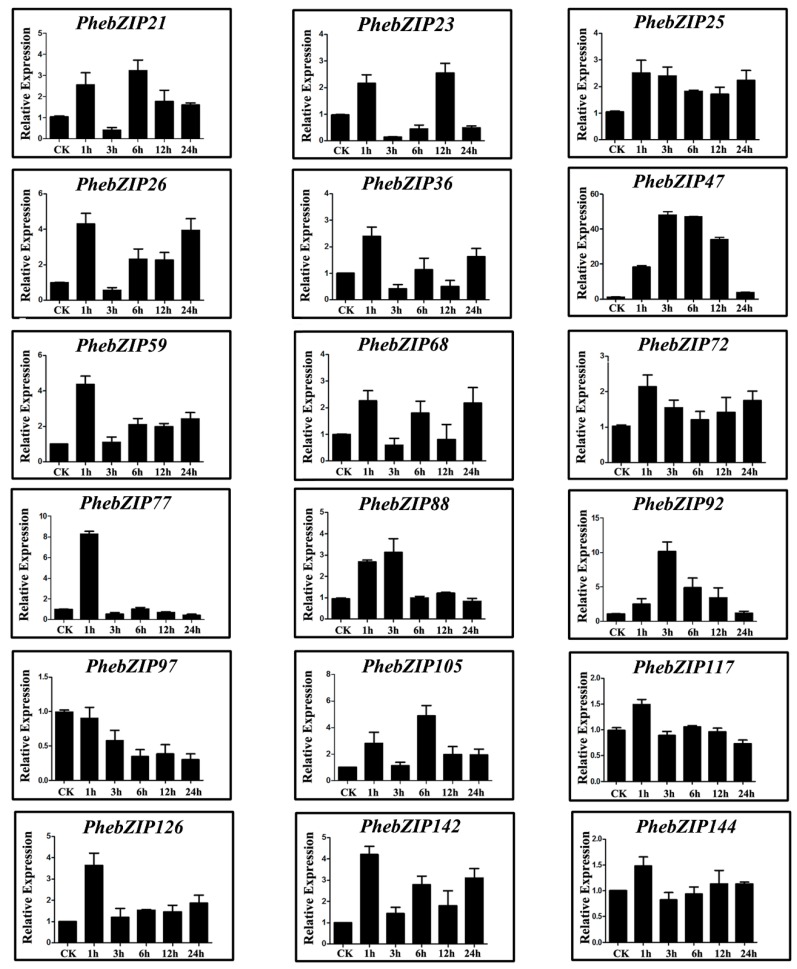
qRT-PCR expression analysis of selected *PhebZIP* genes following abscisic acid (ABA) treatment. Sampling occurred 0, 1, 3, 6, 9, 12, and 24 h after treatment and the relative expression levels were analyzed. Untreated sample expression levels = 1. X-axes represent time points after ABA treatment. Y-axes represent relative gene expression values normalized to the reference gene *TIP41*. Bars indicate standard deviations (SD) from three biological replicates.

Under ABA treatment (Figure 8), the relative expression of 7 *PhebZIP* genes (*PhebZIP26, −47*, −*59*, −*77*, −*92*, −*105* and −*142*) was up-regulated, whereas the expression of *PhebZIP97* was down-regulated at all time-points compared with the control. Additionally, the expression level of *PhebZIP26*, *PhebZIP59*, *PhebZIP77* and *PhebZIP142* peaked at 1 h, while that of *PhebZIP92* peaked at 3 h after treatment. Notably, the expression of *PhebZIP105* peaked at 6 h.

**Figure 9:**
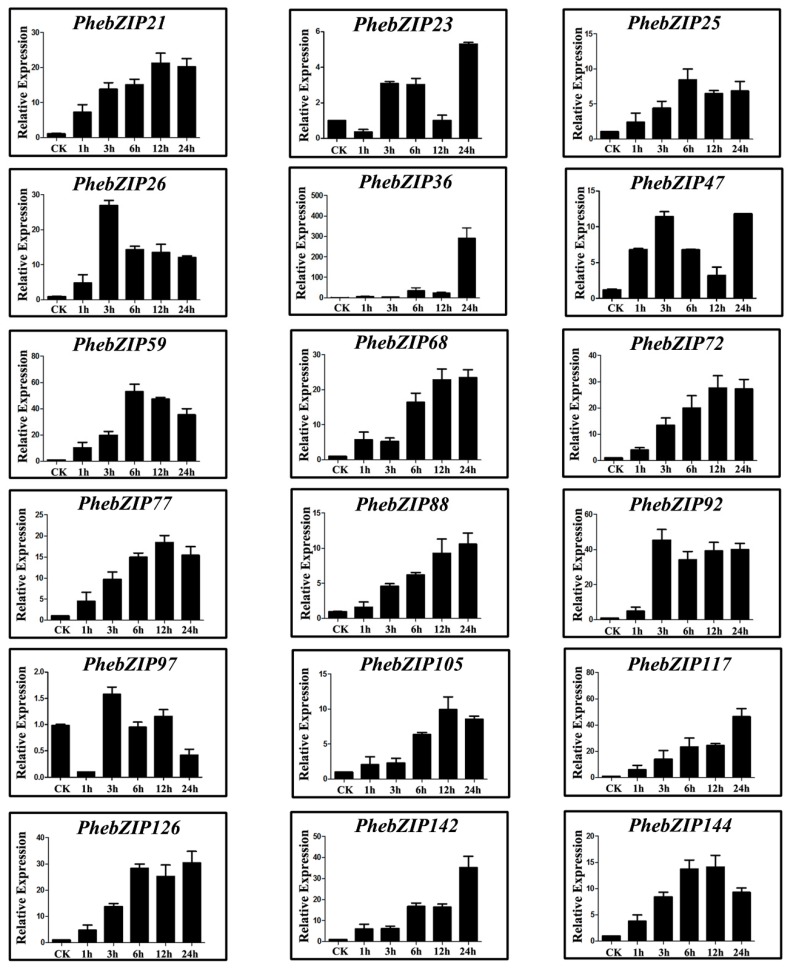
qRT-PCR expression analysis of selected *PhebZIP* genes following gibberellic acid (GA) treatment. The Y-axis indicates the relative expression levels; 0, 1, 3, 6, 9, 12, and 24 (X-axis) indicates hours of treatment. Mean values and standard deviations (SD) were obtained from three biological and three technical replicates.

For the GA treatment, the expression of 16 *PhebZIP* genes (*PhebZIP21*, −*25*, −*26*, −*36*, −*47*, −*59*, −*68*, −*72*, −*77*, −*88*, −*92*, −*105*, −*117*, −*126*, −*142* and −*144*) was found to be up-regulated at each time-point, compared with the control (Figure 9). Additionally, *PhebZIP36* showed the greatest up-regulation (about 300-fold). The paralogous gene pair also showed similar expression patterns in response to GA. Finally, one of these gene (*PhebZIP47*) is up-regulated under all three treatments.

**Figure 10:**
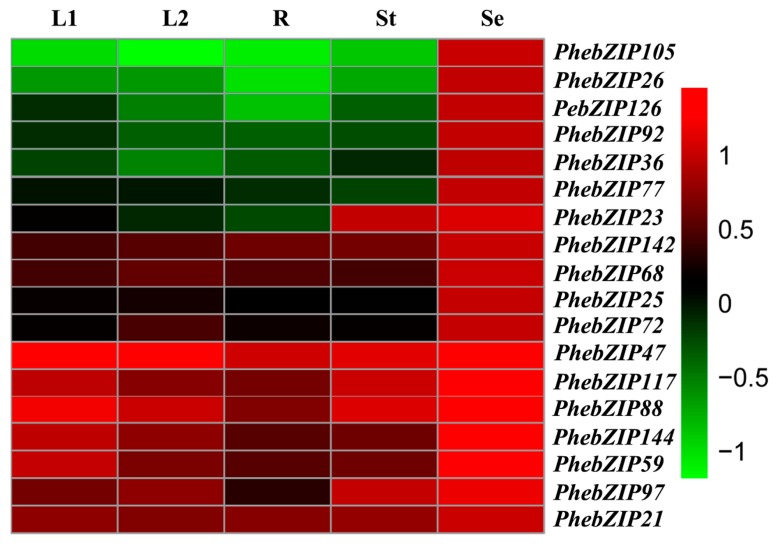
Expression analysis of *PhebZIP* genes across different tissues and developmental stages. Samples were obtained from young leaves, mature leaves, roots, stems, and seeds. Color scale erected vertically at the right side of the picture represents log10 expression values, green indicates lower and red higher transcript abundance. L1: mature leaves; L2: mature leaves; R: roots; St: stems; Se: seeds.

To investigate possible functions of *PhebZIP* genes at different developmental stages or in different organs, we used qRT-PCR to examine the expression profiles of the 18 *PhebZIPs* in young leaves (L1), mature leaves (L2), roots (R), stems (St), and seeds (Se). As shown in Figure 10, nine genes (*PhebZIP21*, −*47*, −*59*, −*68*, −*88*, −*97*, −*117*, −*142* and −*144*) were up-regulated in all tissues, while the remainder showed tissue-specific expression. For instance, all of the genes showed high expression levels in Se and *PhebZIP23* showed high mRNA accumulation in both St and Se. Of the only one pair of paralogous genes, *PhebZIP88* and −*142* showed similar expression patterns in all organs. Beside the paralogues pair, the *PhebZIP 21* and −*47*, *PhebZIP144* and −*59* also had similar expression patterns.

### 2.7. Subcellular Localization and Transactivation Activity Analysis

**Figure 11:**
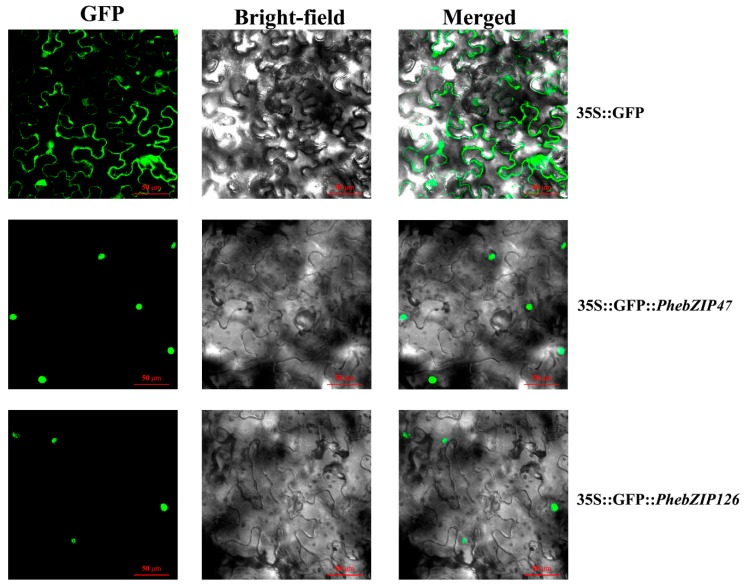
Subcellular localization of *PhebZIP47* and *PhebZIP126.* The two PhebZIP-GFP fusion proteins (*PhebZIP47*-GFP, *PhebZIP126*-GFP) and GFP as a control were transiently expressed in *N. tabacum* leaves and observed under a fluorescence microscope.

For deeper study, we cloned two genes (one was highly expressed in all hormone treatments and the other was only highly expressed under GA treatment) from 18 genes and analyzed their subcellular localization. In addition, the *PhebZIP47* gene of these two genes was highly expressed in all hormone treatments and tissues was selected for transactivation activity analysis. As shown in Figure 11, the control treatment shows green fluorescence throughout the membrane and within the cell nucleus, and the *PhebZIP47*-GFP and *PhebZIP126*-GFP fusion genes’ green fluorescence signals are specifically distributed in the nucleus, consistent with previous predictions that TF binding of specific *cis*-elements occurs in the nucleus.

**Figure 12:**
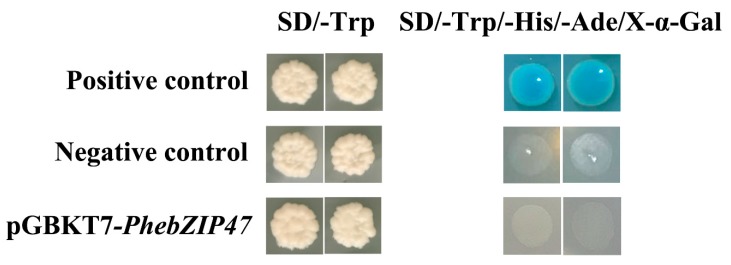
Transactivational analyses of *PhebZIP47* in yeast Y2HGold strain. The positive constructs, negative constructs, and fusion constructs were transformed into yeast Y2HGold strain and successively incubated in SD/-Trp media and SD-His/-Ade/-Trp plate supplemented with X-a-GAL.

All the plasmids were independently transformed into the Y2HGold yeast strain and exhibited visible white colonies on the SD/-Trp medium to explore the transcriptional activity of *PhebZIP47*. The results showed only the positive controls grew well and turned blue in the SD/-Ade/-His/-Trp/X-a-GAL medium. In contrast, the pGBKT7-*PhebZIP47* and negative control group did not grow on this medium (Figure 12), indicating that this gene has no transcriptional activity.

## 3. Discussion

Increasing evidence shows that the bZIP TFs play crucial roles in various developmental and physiological processes and involved in various plant hormone and stress responses in plants. Much of the research into the functions of the bZIP genes have been characterized in model plants, especially in *Arabidopsis thaliana* [3], and some grass family (*Gramineae*) species including *Oryza sativa*, *Sorghum bicolor*, *Zea mays*, *Brachypodium distachyon*, *Triticum aestivum* [4,5,6,7,8]. To our knowledge, however, very little is known about this family in moso bamboo, the only major lineage of grasses. Moso bamboo is one of the most important species for afforestation and timber and has great ecological, cultural, and economic value in the world [34]. Compared with other gramineous plants, research on the function and growth of bamboo has evolved slowly, especially in regard to its response to adverse environmental conditions and plant hormones. Therefore, we want to select a gene family and preliminarily screen some functional genes related to plant growth and development, and stress and hormone response to solve some problems that bamboo will encounter in its growth. The bZIP TF gene family, one of the largest TF families in the eukaryotes, is involved in many biological processes, including plant growth and development, and stress and hormone response. Therefore, we selected this gene family for further analysis.

In this report, we used phylogenetic analysis to classify bZIP TF genes into 10 groups—154 *PhebZIP* genes, along with 89 bZIP genes from rice, and 96 from *Brachypodium distachyon* (Figure 1). We also examined their evolutionary history and selected 18 genes have the higher *cis*-elements under GA, ABA and MeJA stress conditions. For deeper study, two genes were selected for subcellular localization and *PhebZIP47* gene with high expression under three treatments and in five tissues were selected for transactivation activity analysis. The information above provides a comprehensive overview of the bZIP gene family in moso bamboo and lays the foundation for future functional characterization of individual *PhebZIP* genes.

### 3.1. The Distribution and Divergence Times of bZIP Gene Family

Previous research has found that intron numbers of rice and *B. distachyon* are 0–12 and 0–13, respectively [4,7]. And in our research, the intron/exon structural analysis showed that the number of introns in the *PhebZIP* genomic sequences varies from 0 to 17 (Figure 2). This suggested that there were similar gene structure diversity of bZIP genes in different species. Although it has been reported that duplicated genes rarely diverge with respect to their biochemical function; gene duplication is considered to provide the raw material for evolution; duplicated genes may undergo substantial changes in their structures and/or regulatory mechanisms allowing them to assume novel roles [39,40]. The plant specificity is often the result selective gene loss or gain during evolution and can involve chromosomal rearrangements and fusions, such as gene duplication with the three principal types of exon/intron diversification: exon-intron gain/loss, exonization/pseudo-exonization and insertion/deletion [39]. Most of the *PhebZIP* genes in subfamilies I, G, E, F, D and B contain 0–6 introns; the rest subfamilies C, J and H, most of the *PhebZIP* genes have the most introns. The MEME conserved motif analysis shows that motif 17 and 18 are unique to subfamily D, motif 8 is only found in subfamily G, and subfamily F only contains motif 1. In addition, the motifs 1 is present in most of the *PhebZIPs*. Many of the *PhebZIPs* encoded proteins of the same subfamily contain very similar motif compositions.

According to the chromosomal location map (Figure S1), 154 bZIP genes of moso bamboo were distributed irregularly across 23 chromosomes. To estimate evolutionary rates and determine the relative divergence of moso bamboo, the Ks values and Ka/Ks ratios were estimated for the paralogous (*Pe*-*Pe*) gene pairs (Figure 5). The frequency distribution of relative Ks values peaked at 0.1–0.2 (Figure 5A), suggesting that a large-scale genome duplication event occurred around 7–15 MYA creating the moso bamboo bZIP TF gene paralogous pairs. A Ka/Ks value of < 1 indicates that a gene underwent negative or purifying selection, while Ka/Ks =1 and > 1 indicate neutral selection, and positive selection, respectively [41,42]. All the bZIP TF gene paralogue Ka/Ks ratios in moso bamboo are < 1 (Figure 5B), indicating that the *PhebZIP* family paralogous principally underwent purifying selection.

### 3.2. PhebZIP Genes Contain Higher Number of cis-Elements Were Selected for qRT-PCR Analysis

Accumulated evidence has confirmed that many bZIP TFs participate in varied developmental processes, and stress and hormone responses, including abscisic acid (ABA) [22], gibberellin (GA) [23], ethylene [24], pathogen infection [25], drought [26], cold [27]. It is worth noting that gene function and regulation are largely determined by *cis*-regulatory elements [35]. The *cis*-elements analysis showed that the *PhebZIP* gene containing many promoter elements related to hormone and stress response. However, we ultimately selected 18 *PhebZIP* genes containing a large number of promoter elements associated with GA, ABA, and MeJA-response for further analysis by qRT-PCR.

From the *cis*-elements analysis, the gibberellin-responsive *cis*-regulatory elements (GARE motif, P-box, and TATC-box) are seen in 10 of the *PhebZIP* genes. Among these genes, nine genes have one or more elements, and are highly expressed under GA treatment. Only one gene (*PhebZIP97*) contains one element and are not highly expressed under GA treatment. On the contrary, one moso bamboo genes *PhebZIP97* has no CGTCA-motif elements, but is highly expressed after MeJA treatment. This phenomenon suggests that the number of promoters is related to gene expression in respond to adverse conditions but not the only criterion, and the expression induction of genes are complex biological processes.

Moso bamboo will encounter all kinds of adverse environmental conditions during their life. MeJA as a plant hormone and signal molecule related to injury, is widely existed in plants [25]. Exogenous application can stimulate the expression of defense plant genes and induce chemical defense. Under MeJA, which simulated mechanical damage and insect feeding have similar effects, seven of *PhebZIP* genes were significantly up-regulated, suggested that these gene increase resistance to stress. The ABA plays a vital role in plants responding to abiotic stress. In *Arabidopsis*, *ABF2*, *ABF3*, and *ABF4* were significantly up-regulated by ABA. Further functional analyses showed that these genes were involved in the regulation of ABA-mediated processes, such as root elongation and seed germination [43]. In our research, the relative expression showed that seven of selected *PhebZIP* genes to be significantly up-regulated, and one gene was down-regulated after ABA treatment, indicating the comprehensive response of bZIP genes to ABA in moso bamboo. GA, which are tetracyclic diterpenoid growth factors, are essential regulators in many aspects of plant development, including seed germination, stem elongation, and flowering [23]. The expression pattern analysis showed that almost all of the genes we selected were highly expressed under GA treatment. The results suggest that these genes may be largely involved in plant development and growth.

To further understand the expression of these genes in different tissues/organs, growth and development, five organs were selected for qRT-PCR and the result indicated that most of the genes are expressed in these tissues, possibly suggesting that these genes play important roles and have widespread involvement in plant growth and development, and is consistent with previous research in *Arabidopsis* [3]. Simultaneously, the expression pattern analysis of tissues also shows all *PhebZIP* genes to be highly expressed in seeds, which may play a vital role in moso bamboo seed maturation [18]. It is worth noting that the expression patterns of paralogous *PhebZIP* gene pairs under the three hormone treatments and in the five organs are very similar. This further proves that these paralogous gene pairs are functionally similar. In summary, the expression profiles of 18 selected *PhebZIP* genes under GA, ABA, and MeJA and in five organs revealed that these genes are widely involved in growth and development, stress and hormone response.

### 3.3. Subcellular Localization and Transactivation Activity Analysis

In this study, the subcellular localization of *PhebZIP47* and *PhebZIP126* in the nucleus is consistent with previous predictions of bZIP TF binding to *cis*-elements in the nucleus, thus, these two genes are typical nuclear localization transcription factors. It is worth noting that the *PhebZIP47* was highly expressed under three hormone treatments and in all tissues. We want to know if this gene is a transcriptional activator. The transactivation activity analysis showed that *PhebZIP47* had no transcriptional activation activity in yeast. Similar results were observed in rice that *OsbZIP71* has no transcriptional activity in yeast [44]. This phenomenon may be explained by the activation of this protein possibly relying on some posttranslational modifications or needing to be activated by some unknown upstream proteins [45].

## 4. Material and Methods

### 4.1. PhebZIP TF Database Searches

All of the annotated protein sequences from the moso bamboo genome database (http://server.ncgr.ac.cn/bamboo/index.php) were downloaded to identify genomic bZIP TFs [46]. Moreover, to avoid missing any moso bamboo bZIP genes, we used the Hidden Markov Model (HMM) of the bZIP domain (Pfam PF00170) to confirm them in our protein dataset using their Web-based HMM search tool [47,48]. The common domains of the Rice and *B. distachyon* gene families were used in Blastp (e-value = le*^−^*^5^) to identify corresponding moso bamboo gene families. Finally, all non-redundant candidate *PhebZIP* sequence translations were checked against the PFAM (https://pfam.xfam.org) (access in January 2019). databases to confirm the existence of the bZIP domain [49]. Redundant sequences and those without bZIP domains were manually removed from our dataset.

### 4.2. PhebZIP Genes: Phylogenetic Classification, Intron/Exon Structural Analysis, and Conserved Motifs

Rice and *B. distachyon* bZIP protein sequences were obtained from the Rice Genome Annotation Project (http://rice.plantbiology.msu.edu/)(access in January 2019). [50] and *Brachypodium* Genome Database (http://www.plantgdb.org/BdGDB/)(access in January 2019). [7] databases, respectively. Multiple sequence alignments of bZIP proteins from moso bamboo, rice, and *B. distachyon* were performed using the ClustalW program [51]. MEGA6.0 software was used to construct a combined phylogenetic tree (neighbor-joining with 1000 bootstrap replicates) [52]. The *PhebZIP* only gene protein sequence phylogenetic tree was obtained using the same methods. A candidate *PhebZIP* exon/intron gene structure was visualized using the gene structure display server (http://gsds.cbi.pku.edu.cn) (access on in January 2019). [33]. Additionally, the *PhebZIP* encoded protein sequence motifs, which may or may not correspond to previously documented motifs, were identified using MEME online (http://meme-suite.org/tools/meme) (access in January 2019). with our *PhebZIP* protein sequence alignment [53].

### 4.3. Chromosomal Location

Based on chromosomal position information provided by the ncgr database (http://server.ncgr.ac.cn/bamboo/index.php) (access in January 2019), a chromosomal location image for the *PhebZIP* genes was obtained using the MapInspect software (http://www. plantbreeding.wur.nl/uk/software_mapinspect.html) (access in January 2019).

### 4.4. Microsynteny and Evolutionary Patterns of the bZIP Genes in moso bamboo

The duplication of bZIP genes were identified using MCScanX (http://chibba.pgml.uga.edu/mcscan2/) (access in January 2019). [54]. First, whole-genome protein sequences from moso bamboo were searched using BLASTP with an E-value cutoff of 1e^−10^, and identity > 50%. Then, the default parameters of MCScanX were used for detecting the synteny regions. Finally, based on information about collinear pairs and genetic location, circos was used to create collinear analysis diagrams [55]. The selective pressure and the occurrence of duplication events were estimated on the datasets by calculating non-synonymous (Ka) substitution and synonymous (Ks) rates between the collinear pairs. A synonymous substitution (Ks) is defined as a mutation in which a nucleotide base is replaced by a different base in a protein-coding region of a gene that does not result in an amino acid change in the encoded protein, while a non-synonymous substitution (Ka) results in a change in the amino acid sequence of a protein [56]. The non-synonymous and synonymous substitution rates were then calculated using DnaSP 5 to analyze gene duplication events [57,58]. The Ks value was used as a proxy to estimate the timing of large-scale duplication events [59], with Ks converted into a time framework using the formula T = Ks/2λ × 10^−6^ Mya [60]. The clock-like synonymous substitution rate variable (λ) was specified at 6.5 × 10^−9^ years, as per previous estimates [46].

### 4.5. Analysis of PhebZIP TF Putative Promoter Regions

We examined 2000-bp regions upstream of the transcriptional start in all of our candidate *PhebZIP* sequences to identify putative *cis*-elements in these promoter regions. We used the Web-based promoter search Plant-CARE (http://bioinformatics.psb.ugent.be/webtools/plantcare/html/) (access in January 2019). [61] to identify those putative cis-acting elements related to stress responses, hormone effects.

### 4.6. Plant Materials and Phytohormone Treatments

Moso bamboo seeds were identified and provided by the Guilin Forestry Bureau, Guilin, Guang Xi Province, China, and were grown in plastic containers in a greenhouse under a 14-h-light/10-h-dark photoperiod at 25 ± 2 °C and 60–80% humidity. For phytohormone treatments, the young leaves of three-month-old moso bamboo seedlings were sprayed with 10 μM ABA, 100 μM GA, and 100 μM MeJA, separately. All samples were collected at 0, 1, 3, 6, 12, and 24 h separately from seedlings after stress application and untreated leaves (0 h) were used as a control. To obtain tissue-specific expression profiles of *PhebZIP* genes, the tissues of young leaves, mature leaves, roots, stems, and seeds from seedlings were also collected. All plant samples were immediately frozen in liquid N_2_ and stored at −80 °C for RNA extraction. Three biological and three technical replicates were performed to reduce experimental error.

### 4.7. RNA Extraction and qRT-PCR Analysis

Total moso bamboo leaf RNA under different stresses, and from different organs, was extracted with TRIzol reagent (Invitrogen, NH, USA) according to the manufacturer’s instructions. The primer pairs for the 18 selected *PhebZIP* genes were designed using Primer Express 3.0, and the specificity of primers was checked using BLAST searches against a coding sequence (CDS) database downloaded from the moso bamboo genome database (http://server.ncgr.ac.cn/bamboo/index.php) (access in January 2019). [46]. The tonoplast intrinsic protein 41 (*TIP41*) gene, which is stably expressed in moso bamboo, was used as a reference to normalize expression data [62]. The qRT-PCR amplifications using SYBR Green Master Mix reagent (Applied Biosystems) were performed on an ABI 7300 Real-Time system (Applied Biosystems) in 20 μL reactions containing 1 μL of each gene-specific primer, 1 μL of cDNA sample, 7 μL ddH_2_O, and 10 μL SYBR Green Master Mix reagent (Applied Biosystems) [63]. All primers for amplification of *PhebZIP* genes are given in Table S5. The qRT-PCR amplification conditions were: 95 °C for 30 s, followed by 40 cycles of 95 °C for 10 s, 55 ° C for 15 s, and 72 °C for 10 s. A melting curve analysis was performed to determine those primers with the highest specificity and efficiency. The relative expression levels were assessed based on the 2^−ΔΔCT^ method [64].

### 4.8. Subcellular Localization Analysis of PhebZIP47 and PhebZIP126

The full-length *PhebZIP47* and *PhebZIP126* coding sequences were cloned and inserted into the pCAMBIAI1305 vector (TaKaRa, Beijing, China), which contains a *GFP* gene. The constructed *PhebZIP47*-GFP and *PhebZIP126*-GFP vectors were then separately transformed into *Agrobacterium tumefaciens* EHA105 (BIO-RAD, Hercules, CA, USA), and syringes were used to inject the suspensions into *N. tabacum* (tobacco) leaves. The *PhebZIP47*-GFP and *PhebZIP126*-GFP expressions were observed using confocal laser scanning microscopy (CarlZeiss LSM710).

### 4.9. Transactivation Activity Analysis of PhebZIP47

The transactivation activity analysis of *PhebZIP47* protein in yeast was determined as per the previously described method [65]. DNA fragments containing the whole open reading frames of *PhebZIP47* was cloned and then inserted into the pGBKT7 vector (Clontech, Palo Alto, CA, USA) to create the pGBKT7-*PhebZIP47* construct. Then, the recombinant vector, the negative control pGBKT7 plasmids and the positive control pGBKT7-53+pGADT7-T were used to transform the AH109 yeast strain according to the manufacturer’s protocol (Stratagene, Santa Clara, CA, USA). The transformed strains were grown on SD/-Trp and SD/-Trp/-His/-Ade/X-a-Gal plates at 30 °C for 3–5 days.

### 4.10. Ethics Approval and Consent to Participate

The seeds of moso bamboo were collected from Guilin in Guang Xi Province, China in June, 2018. And the seeds were provided and identified by the Guilin Forestry Bureau. All the materials of moso bamboo used and analyzed were public and available for non-commercial purposes. This article did not contain any studies with human participants or animals performed by any of the authors.

### 4.11. Availability of Data and Materials

The genome sequences of moso bamboo, rice and *Brachypodium distachyon* were obtained from the moso bamboo NCGR server (http://server.ncgr.ac.cn/bamboo/index.php) (access in January 2019). Rice Genome Annotation Project database (http://rice.plantbiology.msu.edu) (access in January 2019) and Phytozome database (https://phytozome.jgi.doe.gov) (access in January 2019)., respectively. Moso bamboo bZIP gene IDs were listed in Table S1. The IDs of rice and *Brachypodium distachyon* bZIP gene were exhibited in Table S2.

## 5. Conclusions

Our results represent the first genome-wide identification and analysis of the bZIP TF family in moso bamboo. This includes phylogenetic tree, protein motif prediction, gene structure, microsynteny and evolutionary patterns, all of which suggested a complex evolutionary history of this family in moso bamboo. The expression profile analyses of 18 selected *PhebZIP* genes demonstrate that these genes play pivotal roles in many processes, including stress response, hormone signaling, and in plant growth and development. This research provides a comprehensive understanding of *PhebZIP* genes and may aid in the selection of appropriate candidate genes for further cloning and functional analysis in moso bamboo growth and development and improve their resistance to biological and abiotic stress.

## Supplementary Materials

Supplementary materials can be found at https://www.mdpi.com/1422-0067/20/9/2203/s1.

## Abbreviations

bZIP	Basic leucine zipper
TFs	Transcription factors
GA	Gibberellin
ABA	Abscisic acid
MeJA	Methyl jasmonate
MYA	Million years ago;
qRT-PCR	Quantitative real-time reverse transcription PCR
TIP	Tonoplast intrinsic protein
