# Supplementary material for: Genome-Wide Identification and Expression Analyses of the bZIP Transcription Factor Genes in moso bamboo (Phyllostachys edulis)
Source: Int J Mol Sci. 2019 May 5;20(9):2203. doi: 10.3390/ijms20092203 (PMC6539497; doi:10.3390/ijms20092203)
Supplement: Supplementary file 1 [file ijms-20-02203-s001.zip › Supplementary/Table S1.docx]

**Supplementary Table S1.** Gene names, genomic information, and predicted protein parameters for the 154 putative bZIPgenes in the moso bamboo genome

| Name | GENE ID | chr | Genomic location | ORF length (bp) | Number of amino acids (aa) | PI | MW (Da) | Subcellular location prediction |
| --- | --- | --- | --- | --- | --- | --- | --- | --- |
| *PhebZIP1* | PH02Gene11441 | scaffold_2 | 33440799-33442998(+stand) | 1152 | 383 | 7.67 | 40385.22 | nucl |
| *PhebZIP2* | PH02Gene11469 | scaffold_2 | 34530510-34544859(+stand) | 1875 | 624 | 5.79 | 68545.36 | cyto |
| *PhebZIP3* | PH02Gene33889 | scaffold_2 | 33097045-33105214(-stand) | 1449 | 482 | 6.21 | 52850.09 | nucl |
| *PhebZIP4* | PH02Gene02526 | scaffold_3 | 95522448-95526137(-stand) | 1026 | 341 | 9.59 | 36905.47 | nucl |
| *PhebZIP5* | PH02Gene07171 | scaffold_3 | 8964474-8974094(+stand) | 1125 | 374 | 6.32 | 40083.91 | nucl |
| *PhebZIP6* | PH02Gene07196 | scaffold_3 | 8342272-8344672(-stand) | 1404 | 467 | 9.34 | 50094.59 | nucl |
| *PhebZIP7* | PH02Gene14669 | scaffold_3 | 5585636-5586581(-stand) | 537 | 178 | 9.53 | 18803.01 | nucl |
| *PhebZIP8* | PH02Gene16456 | scaffold_3 | 90137902-90138432(+stand) | 531 | 176 | 8.55 | 20323.79 | nucl |
| *PhebZIP9* | PH02Gene17660 | scaffold_3 | 12011348-12019259(+stand) | 1434 | 477 | 6.84 | 53052.89 | nucl |
| *PhebZIP10* | PH02Gene27949 | scaffold_3 | 17147946-17150043(-stand) | 1116 | 371 | 8.52 | 39757.01 | nucl |
| *PhebZIP11* | PH02Gene42216 | scaffold_3 | 37321825-37322683(-stand) | 492 | 163 | 9.23 | 18206.57 | nucl |
| *PhebZIP12* | PH02Gene43974 | scaffold_3 | 40457901-40462094(+stand) | 891 | 296 | 9.26 | 32851.41 | nucl |
| *PhebZIP13* | PH02Gene17979 | scaffold_4 | 1947293-1954510(+stand) | 1122 | 373 | 6.43 | 39810.64 | nucl |
| *PhebZIP14* | PH02Gene23712 | scaffold_4 | 50367286-50370105(+stand) | 942 | 313 | 5.7 | 33688.83 | nucl |
| *PhebZIP15* | PH02Gene23713 | scaffold_4 | 50382181-50383204(+stand) | 243 | 80 | 10.37 | 8679.74 | nucl |
| *PhebZIP16* | PH02Gene34569 | scaffold_4 | 45454341-45459183(-stand) | 1122 | 373 | 6.1 | 38712.43 | nucl |
| *PhebZIP17* | PH02Gene45429 | scaffold_4 | 5028428-5031017(-stand) | 1707 | 568 | 5.8 | 60245.99 | E.R. |
| *PhebZIP18* | PH02Gene46288 | scaffold_5 | 50235284-50236453(-stand) | 981 | 326 | 7.81 | 35242.55 | nucl |
| *PhebZIP19* | PH02Gene05301 | scaffold_6 | 21908515-21911722(-stand) | 1305 | 434 | 7.86 | 47264.43 | nucl |
| *PhebZIP20* | PH02Gene13695 | scaffold_6 | 38053814-38055304(-stand) | 528 | 175 | 7.84 | 19089.22 | nucl |
| *PhebZIP21* | PH02Gene25237 | scaffold_6 | 42036113-42038604(-stand) | 489 | 162 | 9.8 | 17668.2 | nucl |
| *PhebZIP22* | PH02Gene25658 | scaffold_6 | 39521470-39529050(+stand) | 1317 | 438 | 9.05 | 48585.4 | nucl |
| *PhebZIP23* | PH02Gene30185 | scaffold_6 | 16186873-16189401(+stand) | 1053 | 350 | 6.93 | 37787.99 | nucl |
| *PhebZIP24* | PH02Gene31918 | scaffold_6 | 72793230-72794036(-stand) | 807 | 268 | 5.81 | 28810.41 | nucl |
| *PhebZIP25* | PH02Gene31929 | scaffold_6 | 73121365-73123721(-stand) | 552 | 183 | 8.98 | 20016.5 | nucl |
| *PhebZIP26* | PH02Gene35657 | scaffold_6 | 32808149-32809823(+stand) | 873 | 290 | 5.75 | 31283.09 | nucl |
| *PhebZIP27* | PH02Gene46541 | scaffold_6 | 38824728-38826081(-stand) | 495 | 164 | 5.07 | 17740.17 | nucl |
| *PhebZIP28* | PH02Gene46542 | scaffold_6 | 38832028-38833870(-stand) | 888 | 295 | 4.7 | 31999.2 | nucl |
| *PhebZIP29* | PH02Gene02360 | scaffold_7 | 37281146-37282104(-stand) | 603 | 200 | 5.78 | 22114.02 | nucl |
| *PhebZIP30* | PH02Gene02402 | scaffold_7 | 36257216-36260917(+stand) | 987 | 328 | 9.15 | 35804.3 | nucl |
| *PhebZIP31* | PH02Gene22228 | scaffold_7 | 48707223-48710260(-stand) | 1182 | 393 | 8.93 | 42734.36 | nucl |
| *PhebZIP32* | PH02Gene22743 | scaffold_7 | 23896263-23899174(-stand) | 1941 | 646 | 8.96 | 68722.39 | E.R. |
| *PhebZIP33* | PH02Gene24781 | scaffold_7 | 28125048-28128902(+stand) | 747 | 248 | 6.1 | 26654.75 | nucl |
| *PhebZIP34* | PH02Gene44830 | scaffold_7 | 28991416-28996679(+stand) | 1521 | 506 | 5.99 | 56167.42 | nucl |
| *PhebZIP35* | PH02Gene11238 | scaffold_8 | 63208648-63209520(+stand) | 672 | 223 | 9.67 | 23480.27 | nucl |
| *PhebZIP36* | PH02Gene21911 | scaffold_8 | 27977191-27977787(-stand) | 468 | 155 | 10.18 | 17748.09 | nucl |
| *PhebZIP37* | PH02Gene24830 | scaffold_8 | 78618374-78625161(-stand) | 477 | 158 | 9 | 17414.82 | nucl |
| *PhebZIP38* | PH02Gene28154 | scaffold_8 | 26572488-26575463(-stand) | 1092 | 363 | 8.99 | 40133.5 | nucl |
| *PhebZIP39* | PH02Gene31690 | scaffold_8 | 70510980-70513138(-stand) | 870 | 289 | 5.76 | 31162.78 | nucl |
| *PhebZIP40* | PH02Gene44454 | scaffold_8 | 61435385-61441082(-stand) | 1329 | 442 | 9.06 | 48593.22 | nucl |
| *PhebZIP41* | PH02Gene04216 | scaffold_9 | 62334911-62354570(+stand) | 1212 | 403 | 9.04 | 43271.66 | nucl |
| *PhebZIP42* | PH02Gene05387 | scaffold_9 | 44312326-44328618(+stand) | 1413 | 470 | 5.76 | 52244.86 | nucl |
| *PhebZIP43* | PH02Gene05570 | scaffold_9 | 51559578-51567683(+stand) | 987 | 328 | 6.1 | 35864.16 | nucl |
| *PhebZIP44* | PH02Gene05595 | scaffold_9 | 52051983-52053746(+stand) | 876 | 291 | 6.02 | 32446.76 | nucl |
| *PhebZIP45* | PH02Gene21340 | scaffold_9 | 39027989-39037695(-stand) | 1938 | 645 | 8.45 | 68748.3 | E.R. |
| *PhebZIP46* | PH02Gene24073 | scaffold_9 | 43324560-43327213(+stand) | 750 | 249 | 6.69 | 26504.66 | nucl |
| *PhebZIP47* | PH02Gene25223 | scaffold_9 | 3818884-3819333(+stand) | 327 | 108 | 10.21 | 12074.43 | nucl |
| *PhebZIP48* | PH02Gene04566 | scaffold_10 | 36400510-36405050(+stand) | 1194 | 397 | 5.69 | 41285.24 | nucl |
| *PhebZIP49* | PH02Gene20301 | scaffold_10 | 31856938-31861844(-stand) | 1359 | 452 | 5.61 | 47767.45 | nucl |
| *PhebZIP50* | PH02Gene28254 | scaffold_10 | 24115702-24116229(+stand) | 381 | 126 | 9.28 | 13720.44 | nucl |
| *PhebZIP51* | PH02Gene49718 | scaffold_10 | 3303737-3306310(-stand) | 1710 | 569 | 5.86 | 60467.29 | E.R. |
| *PhebZIP52* | PH02Gene18009 | scaffold_11 | 43547572-43548078(-stand) | 333 | 110 | 11.71 | 11906.8 | chlo |
| *PhebZIP53* | PH02Gene26454 | scaffold_11 | 8195487-8198380(+stand) | 981 | 326 | 6.6 | 34843.68 | nucl |
| *PhebZIP54* | PH02Gene36736 | scaffold_11 | 16339961-16349434(-stand) | 1122 | 373 | 8.43 | 39767.99 | nucl |
| *PhebZIP55* | PH02Gene37785 | scaffold_11 | 5856160-5864979(-stand) | 1458 | 485 | 8.32 | 53367.89 | nucl |
| *PhebZIP56* | PH02Gene44574 | scaffold_11 | 6176933-6178101(+stand) | 528 | 175 | 6.53 | 18958.23 | nucl |
| *PhebZIP57* | PH02Gene07389 | scaffold_11 | 33868767-33869896(-stand) | 534 | 177 | 6.92 | 20116.56 | nucl |
| *PhebZIP58* | PH02Gene09241 | scaffold_12 | 44976945-44999130(+stand) | 858 | 285 | 5.73 | 31134.74 | nucl |
| *PhebZIP59* | PH02Gene12379 | scaffold_12 | 39638958-39640046(+stand) | 480 | 159 | 6.92 | 17732.96 | nucl |
| *PhebZIP60* | PH02Gene22676 | scaffold_12 | 8250482-8255482(+stand) | 984 | 327 | 6.32 | 35135.1 | nucl |
| *PhebZIP61* | PH02Gene39181 | scaffold_12 | 5835182-5840570(-stand) | 1458 | 485 | 6.71 | 53275.63 | nucl |
| *PhebZIP62* | PH02Gene43751 | scaffold_12 | 16924482-16929730(-stand) | 1179 | 392 | 6.34 | 41751.81 | nucl |
| *PhebZIP63* | PH02Gene16163 | scaffold_13 | 90073024-90076345(+stand) | 1389 | 462 | 6.78 | 49433.97 | nucl |
| *PhebZIP64* | PH02Gene19255 | scaffold_13 | 59337000-59346854(-stand) | 1071 | 356 | 8.58 | 37318.27 | nucl |
| *PhebZIP65* | PH02Gene22360 | scaffold_13 | 5695997-5705913(+stand) | 1020 | 339 | 6.56 | 35701.51 | nucl |
| *PhebZIP66* | PH02Gene22559 | scaffold_13 | 131791042-131793149(-stand) | 1188 | 395 | 6.47 | 42873.97 | nucl |
| *PhebZIP67* | PH02Gene24410 | scaffold_13 | 74681438-74682860(-stand) | 582 | 193 | 9.47 | 21833 | vacu |
| *PhebZIP68* | PH02Gene26001 | scaffold_13 | 4706762-4709199(+stand) | 1155 | 384 | 7.15 | 40375.2 | nucl |
| *PhebZIP69* | PH02Gene26016 | scaffold_13 | 4227945-4234131(-stand) | 1452 | 483 | 6.71 | 53264.83 | nucl |
| *PhebZIP70* | PH02Gene28101 | scaffold_13 | 2168135-2169812(-stand) | 705 | 234 | 8.71 | 25436.05 | chlo |
| *PhebZIP71* | PH02Gene35433 | scaffold_13 | 32971023-32971800(+stand) | 429 | 142 | 10.05 | 16851.4 | pero |
| *PhebZIP72* | PH02Gene45256 | scaffold_13 | 2856277-2860373(-stand) | 939 | 312 | 6.47 | 34366.68 | nucl |
| *PhebZIP73* | PH02Gene03743 | scaffold_14 | 65587962-65592560(-stand) | 999 | 332 | 6.13 | 36368.76 | nucl |
| *PhebZIP74* | PH02Gene07659 | scaffold_14 | 45011990-45018804(-stand) | 1098 | 365 | 7.11 | 39225.71 | nucl |
| *PhebZIP75* | PH02Gene10639 | scaffold_14 | 98454359-98455147(+stand) | 585 | 194 | 9.81 | 20549.43 | nucl |
| *PhebZIP76* | PH02Gene17568 | scaffold_14 | 58037613-58042423(+stand) | 816 | 271 | 6.32 | 29429.66 | nucl |
| *PhebZIP77* | PH02Gene31972 | scaffold_14 | 92743279-92744602(+stand) | 1110 | 369 | 6.38 | 40226.85 | nucl |
| *PhebZIP78* | PH02Gene33148 | scaffold_14 | 72186353-72192422(-stand) | 1563 | 520 | 6.47 | 57397.48 | nucl |
| *PhebZIP79* | PH02Gene33151 | scaffold_14 | 72125538-72126588(+stand) | 705 | 234 | 9.09 | 25203.83 | nucl |
| *PhebZIP80* | PH02Gene49751 | scaffold_14 | 64405655-64411300(-stand) | 1377 | 458 | 9.4 | 49851.35 | chlo |
| *PhebZIP81* | PH02Gene05487 | scaffold_15 | 68936553-68938608(+stand) | 468 | 155 | 9.83 | 17056.56 | nucl |
| *PhebZIP82* | PH02Gene07798 | scaffold_15 | 49468992-49469391(-stand) | 330 | 109 | 7.78 | 12193.02 | mito |
| *PhebZIP83* | PH02Gene07819 | scaffold_15 | 47560323-47564974(+stand) | 1005 | 334 | 8.9 | 37197.08 | nucl |
| *PhebZIP84* | PH02Gene11853 | scaffold_15 | 94666532-94670246(-stand) | 1233 | 410 | 5.06 | 42736.05 | nucl |
| *PhebZIP85* | PH02Gene18120 | scaffold_15 | 46990274-47000155(+stand) | 1815 | 604 | 9.11 | 64854.18 | nucl |
| *PhebZIP86* | PH02Gene19131 | scaffold_15 | 58911161-58921051(+stand) | 1830 | 609 | 9.1 | 64682.65 | nucl |
| *PhebZIP87* | PH02Gene20489 | scaffold_15 | 6226508-6228002(+stand) | 1287 | 428 | 9 | 46156.37 | chlo |
| *PhebZIP88* | PH02Gene29194 | scaffold_15 | 96669404-96671972(+stand) | 960 | 319 | 5.73 | 33589.78 | nucl |
| *PhebZIP89* | PH02Gene45188 | scaffold_15 | 44658608-44662783(-stand) | 1050 | 349 | 6.27 | 37582.97 | chlo |
| *PhebZIP90* | PH02Gene04494 | scaffold_16 | 24431202-24434175(+stand) | 924 | 307 | 8.51 | 34073.45 | nucl |
| *PhebZIP91* | PH02Gene04519 | scaffold_16 | 25248548-25252913(+stand) | 1461 | 486 | 8.33 | 53603.15 | nucl |
| *PhebZIP92* | PH02Gene13173 | scaffold_16 | 72607833-72610097(-stand) | 366 | 121 | 9.86 | 13096.87 | nucl |
| *PhebZIP93* | PH02Gene14503 | scaffold_16 | 49124045-49124569(+stand) | 336 | 111 | 5.14 | 12077.49 | nucl |
| *PhebZIP94* | PH02Gene15030 | scaffold_16 | 14767797-14770120(+stand) | 954 | 317 | 6.67 | 33490 | nucl |
| *PhebZIP95* | PH02Gene15488 | scaffold_16 | 16497561-16504098(+stand) | 1563 | 520 | 6.5 | 57198.28 | nucl |
| *PhebZIP96* | PH02Gene15491 | scaffold_16 | 16590892-16592413(+stand) | 1176 | 391 | 8.78 | 41440.05 | nucl |
| *PhebZIP97* | PH02Gene16035 | scaffold_16 | 94521497-94527442(-stand) | 993 | 330 | 7.74 | 36974.61 | nucl |
| *PhebZIP98* | PH02Gene20141 | scaffold_16 | 15674964-15692032(+stand) | 777 | 258 | 6.69 | 27504.13 | nucl |
| *PhebZIP99* | PH02Gene28857 | scaffold_16 | 26184530-26185297(-stand) | 768 | 255 | 6.26 | 28716.78 | cyto |
| *PhebZIP100* | PH02Gene29106 | scaffold_16 | 113848934-113849712(+stand) | 573 | 190 | 9.85 | 20164.06 | nucl |
| *PhebZIP101* | PH02Gene42818 | scaffold_16 | 107090900-107092174(+stand) | 1176 | 391 | 6.07 | 43077.11 | nucl |
| *PhebZIP102* | PH02Gene43362 | scaffold_16 | 43594928-43602090(+stand) | 1209 | 402 | 9.83 | 43074.15 | E.R. |
| *PhebZIP103* | PH02Gene44421 | scaffold_16 | 33228535-33231032(-stand) | 831 | 276 | 6.13 | 29815.99 | nucl |
| *PhebZIP104* | PH02Gene08137 | scaffold_17 | 14954536-14955045(-stand) | 357 | 118 | 9.24 | 14052.84 | nucl |
| *PhebZIP105* | PH02Gene09091 | scaffold_17 | 91334086-91336600(-stand) | 915 | 304 | 5.61 | 32752.6 | nucl |
| *PhebZIP106* | PH02Gene10312 | scaffold_17 | 87299393-87311143(-stand) | 1386 | 461 | 8.88 | 50186.55 | nucl |
| *PhebZIP107* | PH02Gene10795 | scaffold_17 | 8885361-8891741(+stand) | 1020 | 339 | 9.83 | 36627.11 | nucl |
| *PhebZIP108* | PH02Gene31423 | scaffold_17 | 77765130-77766921(-stand) | 759 | 252 | 5.91 | 27057.56 | nucl |
| *PhebZIP109* | PH02Gene34090 | scaffold_17 | 91292893-91295428(-stand) | 921 | 306 | 5.2 | 32692.35 | nucl |
| *PhebZIP110* | PH02Gene34092 | scaffold_17 | 91256184-91258672(-stand) | 897 | 298 | 5.64 | 32068.82 | nucl |
| *PhebZIP111* | PH02Gene41822 | scaffold_17 | 97028317-97032333(+stand) | 585 | 194 | 9.8 | 20668.41 | nucl |
| *PhebZIP112* | PH02Gene04355 | scaffold_18 | 3070055-3070749(+stand) | 558 | 185 | 9.89 | 20729.52 | nucl |
| *PhebZIP113* | PH02Gene04609 | scaffold_18 | 32319643-32333245(+stand) | 1335 | 444 | 6.47 | 49591.27 | nucl |
| *PhebZIP114* | PH02Gene04714 | scaffold_18 | 28309571-28312822(-stand) | 1116 | 371 | 6.57 | 39870.74 | nucl |
| *PhebZIP115* | PH02Gene05093 | scaffold_18 | 24280346-24281073(-stand) | 624 | 207 | 9.17 | 22064.58 | nucl |
| *PhebZIP116* | PH02Gene21644 | scaffold_18 | 37747034-37754957(-stand) | 1086 | 361 | 7.71 | 38642.91 | nucl |
| *PhebZIP117* | PH02Gene23091 | scaffold_18 | 27155907-27158348(-stand) | 1416 | 471 | 9.29 | 50384.68 | nucl |
| *PhebZIP118* | PH02Gene31633 | scaffold_18 | 18691988-18693930(-stand) | 1149 | 382 | 6.57 | 41826.25 | nucl |
| *PhebZIP119* | PH02Gene06509 | scaffold_19 | 10923746-10925964(-stand) | 633 | 210 | 9.36 | 23009 | nucl |
| *PhebZIP120* | PH02Gene20935 | scaffold_19 | 9680907-9685206(-stand) | 1746 | 581 | 6.28 | 61290.84 | nucl |
| *PhebZIP121* | PH02Gene27620 | scaffold_19 | 26536521-26545746(-stand) | 1128 | 375 | 6.23 | 40336.33 | chlo |
| *PhebZIP122* | PH02Gene28656 | scaffold_19 | 27862707-27864289(+stand) | 690 | 229 | 9.4 | 25105.26 | nucl |
| *PhebZIP123* | PH02Gene28667 | scaffold_19 | 28135114-28138051(+stand) | 1005 | 334 | 9.27 | 37340.2 | nucl |
| *PhebZIP124* | PH02Gene32284 | scaffold_19 | 14813369-14824921(-stand) | 1365 | 454 | 8.57 | 50425.39 | nucl |
| *PhebZIP125* | PH02Gene10250 | scaffold_20 | 49624366-49627807(-stand) | 1083 | 360 | 5.16 | 38537.29 | nucl |
| *PhebZIP126* | PH02Gene14383 | scaffold_20 | 48919693-48920224(+stand) | 288 | 95 | 10.67 | 10214.38 | nucl |
| *PhebZIP127* | PH02Gene20776 | scaffold_20 | 28614689-28619736(+stand) | 759 | 252 | 5.59 | 27125.52 | nucl |
| *PhebZIP128* | PH02Gene26852 | scaffold_20 | 43780303-43798632(-stand) | 1023 | 340 | 5.16 | 36649.69 | nucl |
| *PhebZIP129* | PH02Gene34118 | scaffold_20 | 39563523-39565648(+stand) | 834 | 277 | 4.88 | 29677.77 | nucl |
| *PhebZIP130* | PH02Gene35475 | scaffold_20 | 40475385-40478943(+stand) | 660 | 219 | 11.31 | 24439.87 | nucl |
| *PhebZIP131* | PH02Gene40035 | scaffold_20 | 39021518-39041353(-stand) | 1380 | 459 | 8.68 | 50039.56 | nucl |
| *PhebZIP132* | PH02Gene47757 | scaffold_20 | 287100-287868(-stand) | 639 | 212 | 9.81 | 22832.82 | nucl |
| *PhebZIP133* | PH02Gene00867 | scaffold_21 | 110321293-110323593(-stand) | 1242 | 413 | 6.24 | 44387.37 | nucl |
| *PhebZIP134* | PH02Gene00975 | scaffold_21 | 113152476-113162281(-stand) | 2520 | 839 | 5.96 | 91904.76 | cyto |
| *PhebZIP135* | PH02Gene06737 | scaffold_21 | 8757950-8761850(+stand) | 1485 | 494 | 5.63 | 52468.18 | nucl |
| *PhebZIP136* | PH02Gene12419 | scaffold_21 | 70182855-70190914(-stand) | 1047 | 348 | 7.18 | 37660.21 | chlo |
| *PhebZIP137* | PH02Gene13422 | scaffold_21 | 12354837-12358008(+stand) | 1239 | 412 | 9.88 | 44071.01 | nucl |
| *PhebZIP138* | PH02Gene24017 | scaffold_21 | 7556490-7560432(-stand) | 1215 | 404 | 8.49 | 45269.62 | nucl |
| *PhebZIP139* | PH02Gene25703 | scaffold_21 | 78906927-78911262(-stand) | 1005 | 334 | 8.92 | 37255.11 | nucl |
| *PhebZIP140* | PH02Gene25937 | scaffold_21 | 76586342-76587178(-stand) | 543 | 180 | 6.42 | 20582.04 | nucl |
| *PhebZIP141* | PH02Gene26378 | scaffold_21 | 28707021-28707591(+stand) | 489 | 162 | 11.73 | 17371.67 | nucl |
| *PhebZIP142* | PH02Gene31449 | scaffold_21 | 5982171-5984827(-stand) | 1062 | 353 | 6.33 | 37066.71 | nucl |
| *PhebZIP143* | PH02Gene45713 | scaffold_21 | 92475086-92479827(+stand) | 1155 | 384 | 9.44 | 41214.73 | nucl |
| *PhebZIP144* | PH02Gene23643 | scaffold_22 | 4151048-4155039(+stand) | 921 | 306 | 6.26 | 32797.9 | nucl |
| *PhebZIP145* | PH02Gene24093 | scaffold_22 | 1905859-1916020(-stand) | 594 | 197 | 9.85 | 22079.34 | nucl |
| *PhebZIP146* | PH02Gene26159 | scaffold_22 | 12420013-12420681(+stand) | 669 | 222 | 8.9 | 24431.98 | nucl |
| *PhebZIP147* | PH02Gene36693 | scaffold_22 | 30134492-30137555(-stand) | 360 | 119 | 10.74 | 13615.6 | nucl |
| *PhebZIP148* | PH02Gene37677 | scaffold_22 | 3154288-3157555(-stand) | 1383 | 460 | 6.47 | 49580.07 | nucl |
| *PhebZIP149* | PH02Gene47106 | scaffold_22 | 49209097-49217319(+stand) | 1212 | 403 | 6.78 | 44529.46 | nucl |
| *PhebZIP150* | PH02Gene03192 | scaffold_24 | 46258884-46265120(-stand) | 1092 | 363 | 5.95 | 39280.83 | nucl |
| *PhebZIP151* | PH02Gene36596 | scaffold_24 | 65843910-65851165(+stand) | 1179 | 392 | 6.15 | 44147.16 | nucl |
| *PhebZIP152* | PH02Gene41549 | scaffold_24 | 42239829-42241048(-stand) | 639 | 212 | 6.3 | 23524.44 | nucl |
| *PhebZIP153* | PH02Gene41553 | scaffold_24 | 42316434-42316923(-stand) | 330 | 109 | 10.05 | 12152.99 | nucl |
| *PhebZIP154* | PH02Gene49732 | scaffold_35 | 77053-79925(-stand) | 873 | 290 | 5.79 | 31388.56 | golg |
