# Supplementary material for: Genome-Wide Identification and Expression Analyses of the bZIP Transcription Factor Genes in moso bamboo (Phyllostachys edulis)
Source: Int J Mol Sci. 2019 May 5;20(9):2203. doi: 10.3390/ijms20092203 (PMC6539497; doi:10.3390/ijms20092203)
Supplement: Supplementary file 1 [file ijms-20-02203-s001.zip › Supplementary/Table S2.docx]

| **Supplementary Table S2.** Gene names and locus information for the bZIP proteins in rice and *Brachypodium distachyon* | |
| --- | --- |
| **rice** | |
| Gene Name | Gene locus ID |
| *OsbZIP01* | LOC_Os01g07880 |
| *OsbZIP02* | LOC_Os01g11350 |
| *OsbZIP03* | LOC_Os01g17260 |
| *OsbZIP04* | LOC_Os01g36220 |
| *OsbZIP05* | LOC_Os01g46970 |
| *OsbZIP06* | LOC_Os01g55150 |
| *OsbZIP07* | LOC_Os01g58760 |
| *OsbZIP08* | LOC_Os01g59350 |
| *OsbZIP09* | LOC_Os01g59760 |
| *OsbZIP10* | LOC_Os01g64000 |
| *OsbZIP11* | LOC_Os01g64020 |
| *OsbZIP12* | LOC_Os01g64730 |
| *OsbZIP13* | LOC_Os02g03580 |
| *OsbZIP14* | LOC_Os02g03960 |
| *OsbZIP15* | LOC_Os02g07840 |
| *OsbZIP16* | LOC_Os02g09830 |
| *OsbZIP17* | LOC_Os02g10140 |
| *OsbZIP18* | LOC_Os02g10860 |
| *OsbZIP19* | LOC_Os02g14910 |
| *OsbZIP20* | LOC_Os02g16680 |
| *OsbZIP21* | LOC_Os02g33560 |
| *OsbZIP22* | LOC_Os02g49560 |
| *OsbZIP23* | LOC_Os02g52780 |
| *OsbZIP24* | LOC_Os02g58670 |
| *OsbZIP25* | LOC_Os03g03550 |
| *OsbZIP26* | LOC_Os03g13614 |
| *OsbZIP27* | LOC_Os03g19370 |
| *OsbZIP28* | LOC_Os03g20310 |
| *OsbZIP29* | LOC_Os03g20650 |
| *OsbZIP30* | LOC_Os03g21800 |
| *OsbZIP31* | LOC_Os03g47200 |
| *OsbZIP32* | LOC_Os03g56010 |
| *OsbZIP33* | LOC_Os03g58250 |
| *OsbZIP34* | LOC_Os03g59460 |
| *OsbZIP35* | LOC_Os04g10260 |
| *OsbZIP36* | LOC_Os04g41820 |
| *OsbZIP37* | LOC_Os04g54474 |
| *OsbZIP38* | LOC_Os05g03860 |
| *OsbZIP39* | LOC_Os05g34050 |
| *OsbZIP40* | LOC_Os05g36160 |
| *OsbZIP41* | LOC_Os05g37170 |
| *OsbZIP42* | LOC_Os05g41070 |
| *OsbZIP43* | LOC_Os05g41280 |
| *OsbZIP44* | LOC_Os05g41540 |
| *OsbZIP45* | LOC_Os05g49420 |
| *OsbZIP46* | LOC_Os06g10880 |
| *OsbZIP47* | LOC_Os06g15480 |
| *OsbZIP48* | LOC_Os06g39960 |
| *OsbZIP49* | LOC_Os06g41100 |
| *OsbZIP50* | LOC_Os06g41770 |
| *OsbZIP51* | LOC_Os06g42690 |
| *OsbZIP52* | LOC_Os06g45140 |
| *OsbZIP53* | LOC_Os06g50310 |
| *OsbZIP54* | LOC_Os06g50480 |
| *OsbZIP55* | LOC_Os06g50600 |
| *OsbZIP56* | LOC_Os06g50830 |
| *OsbZIP57* | LOC_Os07g03220 |
| *OsbZIP58* | LOC_Os07g08420 |
| *OsbZIP59* | LOC_Os07g10890 |
| *OsbZIP60* | LOC_Os07g44950 |
| *OsbZIP61* | LOC_Os07g48180 |
| *OsbZIP62* | LOC_Os07g48660 |
| *OsbZIP63* | LOC_Os07g48820 |
| *OsbZIP64* | LOC_Os08g07970 |
| *OsbZIP65* | LOC_Os08g26880 |
| *OsbZIP66* | LOC_Os08g36790 |
| *OsbZIP67* | LOC_Os08g38020 |
| *OsbZIP68* | LOC_Os08g43090 |
| *OsbZIP69* | LOC_Os08g43600 |
| *OsbZIP70* | LOC_Os09g10840 |
| *OsbZIP71* | LOC_Os09g13570 |
| *OsbZIP72* | LOC_Os09g28310 |
| *OsbZIP73* | LOC_Os09g29820 |
| *OsbZIP74* | LOC_Os09g31390 |
| *OsbZIP75* | LOC_Os09g34060 |
| *OsbZIP76* | LOC_Os09g34880 |
| *OsbZIP77* | LOC_Os09g36910 |
| *OsbZIP78* | LOC_Os10g38820 |
| *OsbZIP79* | LOC_Os11g05480 |
| *OsbZIP80* | LOC_Os11g05640 |
| *OsbZIP81* | LOC_Os11g06170 |
| *OsbZIP82* | LOC_Os11g11100 |
| *OsbZIP83* | LOC_Os12g05680 |
| *OsbZIP84* | LOC_Os12g06520 |
| *OsbZIP85* | LOC_Os12g09270 |
| *OsbZIP86* | LOC_Os12g13170 |
| *OsbZIP87* | LOC_Os12g37410 |
| *OsbZIP88* | LOC_Os12g40920 |
| *OsbZIP89* | LOC_Os12g43790 |
| ***Brachypodium distachyon*** | |
| Gene Name | Gene locus ID |
| *BdbZIP1* | bradi1g04510 |
| *BdbZIP2* | bradi1g05480 |
| *BdbZIP3* | bradi1g07310 |
| *BdbZIP4* | bradi1g12620 |
| *BdbZIP5* | bradi1g17210 |
| *BdbZIP6* | bradi1g17335 |
| *BdbZIP7* | bradi1g17700 |
| *BdbZIP8* | bradi1g19700 |
| *BdbZIP9* | bradi1g19702 |
| *BdbZIP10* | bradi1g29920 |
| *BdbZIP11* | bradi1g30140 |
| *BdbZIP12* | bradi1g30750 |
| *BdbZIP13* | bradi1g31700 |
| *BdbZIP14* | bradi1g35550 |
| *BdbZIP15* | bradi1g35790 |
| *BdbZIP16* | bradi1g36750 |
| *BdbZIP17* | bradi1g43660 |
| *BdbZIP18* | bradi1g43900 |
| *BdbZIP19* | bradi1g46060 |
| *BdbZIP20* | bradi1g54180 |
| *BdbZIP21* | bradi1g55450 |
| *BdbZIP22* | bradi1g63170 |
| *BdbZIP23* | bradi1g63840 |
| *BdbZIP24* | bradi1g64060 |
| *BdbZIP25* | bradi1g64550 |
| *BdbZIP26* | bradi1g68560 |
| *BdbZIP27* | bradi1g76690 |
| *BdbZIP28* | bradi2g04590 |
| *BdbZIP29* | bradi2g06790 |
| *BdbZIP30* | bradi2g10830 |
| *BdbZIP31* | bradi2g15940 |
| *BdbZIP32* | bradi2g21197 |
| *BdbZIP33* | bradi2g21200 |
| *BdbZIP34* | bradi2g21380 |
| *BdbZIP35* | bradi2g21820 |
| *BdbZIP36* | bradi2g23890 |
| *BdbZIP37* | bradi2g24120 |
| *BdbZIP38* | bradi2g25400 |
| *BdbZIP39* | bradi2g38380 |
| *BdbZIP40* | bradi2g40580 |
| *BdbZIP41* | bradi2g45567 |
| *BdbZIP42* | bradi2g45570 |
| *BdbZIP43* | bradi2g50220 |
| *BdbZIP44* | bradi2g52590 |
| *BdbZIP45* | bradi2g52860 |
| *BdbZIP46* | bradi2g53060 |
| *BdbZIP47* | bradi2g55550 |
| *BdbZIP48* | bradi2g55567 |
| *BdbZIP49* | bradi2g55570 |
| *BdbZIP50* | bradi2g56080 |
| *BdbZIP51* | bradi2g56096 |
| *BdbZIP52* | bradi3g00300 |
| *BdbZIP53* | bradi3g02730 |
| *BdbZIP54* | bradi3g02980 |
| *BdbZIP55* | bradi3g05577 |
| *BdbZIP56* | bradi3g05580 |
| *BdbZIP57* | bradi3g06160 |
| *BdbZIP58* | bradi3g06670 |
| *BdbZIP59* | bradi3g07030 |
| *BdbZIP60* | bradi3g07540 |
| *BdbZIP61* | bradi3g09340 |
| *BdbZIP62* | bradi3g15590 |
| *BdbZIP63* | bradi3g22040 |
| *BdbZIP64* | bradi3g31890 |
| *BdbZIP65* | bradi3g38200 |
| *BdbZIP66* | bradi3g38840 |
| *BdbZIP67* | bradi3g41817 |
| *BdbZIP68* | bradi3g41820 |
| *BdbZIP69* | bradi3g41980 |
| *BdbZIP70* | bradi3g45170 |
| *BdbZIP71* | bradi3g56290 |
| *BdbZIP72* | bradi3g57960 |
| *BdbZIP73* | bradi3g60870 |
| *BdbZIP74* | bradi4g00810 |
| *BdbZIP75* | bradi4g02570 |
| *BdbZIP76* | bradi4g04720 |
| *BdbZIP77* | bradi4g22130 |
| *BdbZIP78* | bradi4g24937 |
| *BdbZIP79* | bradi4g24940 |
| *BdbZIP80* | bradi4g26670 |
| *BdbZIP81* | bradi4g27100 |
| *BdbZIP82* | bradi4g27720 |
| *BdbZIP83* | bradi4g32090 |
| *BdbZIP84* | bradi4g32920 |
| *BdbZIP85* | bradi4g33740 |
| *BdbZIP86* | bradi4g35240 |
| *BdbZIP87* | bradi4g35370 |
| *BdbZIP88* | bradi4g36587 |
| *BdbZIP89* | bradi4g39630 |
| *BdbZIP90* | bradi4g40540 |
| *BdbZIP91* | bradi4g41890 |
| *BdbZIP92* | bradi4g42120 |
| *BdbZIP93* | bradi4g43850 |
| *BdbZIP94* | bradi5g14497 |
| *BdbZIP95* | bradi5g14500 |
| *BdbZIP96* | bradi5g23340 |
