# Supplementary material for: Genome-Wide Identification and Expression Analyses of the bZIP Transcription Factor Genes in moso bamboo (Phyllostachys edulis)
Source: Int J Mol Sci. 2019 May 5;20(9):2203. doi: 10.3390/ijms20092203 (PMC6539497; doi:10.3390/ijms20092203)
Supplement: Supplementary file 1 [file ijms-20-02203-s001.zip › Supplementary/Table S3.docx]

| **Supplementary Table S3.** Nucleotide substitution rates for the paralogous bZIP gene pairs identified in the moso bamboo genome   \| **Paralogous pairs** \| **Ka value** \| **Ks value** \| **Ka/Ks value** \| \| --- \| --- \| --- \| --- \| \| *PhebZIP4-PhebZIP23* \| 0.205288165 \| 0.616357877 \| 0.333066506 \| \| *PhebZIP8-PhebZIP36* \| 0.256144162 \| 1.401589808 \| 0.182752586 \| \| *PhebZIP10-PhebZIP23* \| 0.346751334 \| 0.81375863 \| 0.426110792 \| \| *PhebZIP19-PhebZIP38* \| 0.025832812 \| 0.142488322 \| 0.181297748 \| \| *PhebZIP20-PhebZIP35* \| 0.098509641 \| 0.192921318 \| 0.510620817 \| \| *PhebZIP22-PhebZIP40* \| 0.072451399 \| 0.135871997 \| 0.533232751 \| \| *PhebZIP25-PhebZIP37* \| 0.106507748 \| 0.357631328 \| 0.297814369 \| \| *PhebZIP26-PhebZIP39* \| 0.056408722 \| 0.194271264 \| 0.290360605 \| \| *PhebZIP30-PhebZIP43* \| 0.043039696 \| 0.169073519 \| 0.254562017 \| \| *PhebZIP32-PhebZIP45* \| 0.045441764 \| 0.148212136 \| 0.306599477 \| \| *PhebZIP33-PhebZIP46* \| 0.084151219 \| 0.144809098 \| 0.581118317 \| \| *PhebZIP34-PhebZIP42* \| 0.04951046 \| 0.098387718 \| 0.503217889 \| \| *PhebZIP46-PhebZIP7* \| 0.653524059 \| 2.453386728 \| 0.266376292 \| \| *PhebZIP48-PhebZIP88* \| 0.219915365 \| 0.840930744 \| 0.261514241 \| \| *PhebZIP48-PhebZIP142* \| 0.218359447 \| 0.852360176 \| 0.256182132 \| \| *PhebZIP48-PhebZIP16* \| 0.024805899 \| 0.165996913 \| 0.149435905 \| \| *PhebZIP49-PhebZIP135* \| 0.247421408 \| 0.578811257 \| 0.427464747 \| \| *PhebZIP49-PhebZIP84* \| 0.253522331 \| 0.603869407 \| 0.419829731 \| \| *PhebZIP49-PhebZIP14* \| 0.069991228 \| 0.166791412 \| 0.419633282 \| \| *PhebZIP51-PhebZIP17* \| 0.048009935 \| 0.169753537 \| 0.282821409 \| \| *PhebZIP53-PhebZIP2* \| 0.223890318 \| 0.770056716 \| 0.290745232 \| \| *PhebZIP53-PhebZIP65* \| 0.091345121 \| 0.557106831 \| 0.163963383 \| \| *PhebZIP53-PhebZIP60* \| 0.042999795 \| 0.152915708 \| 0.28119933 \| \| *PhebZIP54-PhebZIP62* \| 0.04111029 \| 0.154742417 \| 0.265669174 \| \| *PhebZIP55-PhebZIP69* \| 0.056335621 \| 0.149210892 \| 0.377557028 \| \| *PhebZIP55-PhebZIP3* \| 0.037303726 \| 0.170573595 \| 0.218695783 \| \| *PhebZIP55-PhebZIP61* \| 0.050423318 \| 0.146020695 \| 0.345316245 \| \| *PhebZIP56-PhebZIP1* \| 0.183281829 \| 0.423128703 \| 0.433158581 \| \| *PhebZIP57-PhebZIP59* \| 0.041667049 \| 0.098135321 \| 0.424587689 \| \| *PhebZIP60-PhebZIP2* \| 0.240948896 \| 0.652194097 \| 0.36944354 \| \| *PhebZIP60-PhebZIP65* \| 0.07920579 \| 0.528755368 \| 0.149796663 \| \| *PhebZIP61-PhebZIP69* \| 0.057026752 \| 0.105215333 \| 0.542000396 \| \| *PhebZIP61-PhebZIP3* \| 0.044610597 \| 0.174479959 \| 0.255677486 \| \| *PhebZIP63-PhebZIP6* \| 0.204230638 \| 0.694979706 \| 0.293865614 \| \| *PhebZIP63-PhebZIP117* \| 0.199992917 \| 0.742740637 \| 0.269263464 \| \| *PhebZIP63-PhebZIP148* \| 0.022669268 \| 0.16717757 \| 0.13559994 \| \| *PhebZIP64-PhebZIP4* \| 0.309993635 \| 0.938103078 \| 0.330447306 \| \| *PhebZIP64-PhebZIP116* \| 0.171632736 \| 0.595784433 \| 0.288078584 \| \| *PhebZIP64-PhebZIP10* \| 0.201903037 \| 0.657541186 \| 0.307057628 \| \| *PhebZIP65-PhebZIP2* \| 0.215054256 \| 0.496335259 \| 0.433284261 \| \| *PhebZIP66-PhebZIP133* \| 0.195920284 \| 0.472705212 \| 0.414466097 \| \| *PhebZIP66-PhebZIP87* \| 0.350735627 \| 0.50678466 \| 0.692080196 \| \| *PhebZIP66-PhebZIP18* \| 0.065050761 \| 0.158673037 \| 0.409967325 \| \| *PhebZIP67-PhebZIP119* \| 0.377831724 \| 0.912236668 \| 0.414181689 \| \| *PhebZIP67-PhebZIP147* \| 0.161905181 \| 0.238202814 \| 0.679694661 \| \| *PhebZIP67-PhebZIP11* \| 0.290970905 \| 0.862552939 \| 0.337336866 \| \| *PhebZIP69-PhebZIP3* \| 0.065641616 \| 0.160478985 \| 0.40903559 \| \| *PhebZIP73-PhebZIP30* \| 0.132265935 \| 0.654168281 \| 0.202189465 \| \| *PhebZIP73-PhebZIP90* \| 0.034809873 \| 0.078214888 \| 0.44505431 \| \| *PhebZIP73-PhebZIP43* \| 0.134322214 \| 0.743095705 \| 0.180760316 \| \| *PhebZIP74-PhebZIP41* \| 0.208868629 \| 0.760000028 \| 0.274827134 \| \| *PhebZIP75-PhebZIP100* \| 0.016552396 \| 0.093566983 \| 0.17690424 \| \| *PhebZIP76-PhebZIP103* \| 0.03007503 \| 0.103565614 \| 0.290395906 \| \| *PhebZIP77-PhebZIP127* \| 0.373378821 \| 1.141996976 \| 0.326952548 \| \| *PhebZIP77-PhebZIP108* \| 0.348569265 \| 1.146234276 \| 0.304099496 \| \| *PhebZIP77-PhebZIP101* \| 0.043916558 \| 0.137935245 \| 0.318385323 \| \| *PhebZIP78-PhebZIP42* \| 0.132108146 \| 0.727709469 \| 0.181539683 \| \| *PhebZIP78-PhebZIP95* \| 0.025293531 \| 0.095411712 \| 0.265098806 \| \| *PhebZIP78-PhebZIP34* \| 0.189427141 \| 0.75343973 \| 0.251416448 \| \| *PhebZIP79-PhebZIP96* \| 0.084985464 \| 0.185199597 \| 0.458885796 \| \| *PhebZIP80-PhebZIP29* \| 0.162506212 \| 1.675394033 \| 0.096995817 \| \| *PhebZIP82-PhebZIP140* \| 0.115669548 \| 0.174564308 \| 0.662618544 \| \| *PhebZIP83-PhebZIP139* \| 0.009171421 \| 0.090710644 \| 0.10110634 \| \| *PhebZIP83-PhebZIP123* \| 0.041142753 \| 0.718505076 \| 0.057261605 \| \| *PhebZIP84-PhebZIP135* \| 0.060696975 \| 0.135196368 \| 0.448954186 \| \| *PhebZIP84-PhebZIP14* \| 0.252821958 \| 0.728575385 \| 0.347008646 \| \| *PhebZIP86-PhebZIP143* \| 0.033851301 \| 0.109070631 \| 0.310361282 \| \| *PhebZIP87-PhebZIP133* \| 0.182257519 \| 0.295681234 \| 0.61639867 \| \| *PhebZIP87-PhebZIP18* \| 0.213593296 \| 0.410412066 \| 0.5204362 \| \| *PhebZIP88-PhebZIP142* \| 0.096311208 \| 0.161390762 \| 0.596757876 \| \| *PhebZIP88-PhebZIP16* \| 0.22848811 \| 0.867346129 \| 0.263433596 \| \| *PhebZIP89-PhebZIP136* \| 0.033848894 \| 0.126339196 \| 0.267920768 \| \| *PhebZIP89-PhebZIP13* \| 0.14652013 \| 0.46206047 \| 0.317101634 \| \| *PhebZIP89-PhebZIP121* \| 0.139447589 \| 0.472616388 \| 0.295054493 \| \| *PhebZIP90-PhebZIP30* \| 0.12439897 \| 0.684309827 \| 0.181787496 \| \| *PhebZIP90-PhebZIP43* \| 0.135623943 \| 0.76415311 \| 0.177482681 \| \| *PhebZIP91-PhebZIP44* \| 0.145810338 \| 0.617382892 \| 0.236174892 \| \| *PhebZIP95-PhebZIP42* \| 0.126211261 \| 0.770468346 \| 0.163811092 \| \| *PhebZIP95-PhebZIP34* \| 0.183198264 \| 0.788722384 \| 0.232272175 \| \| *PhebZIP98-PhebZIP46* \| 0.192216497 \| 0.633561957 \| 0.303390213 \| \| *PhebZIP98-PhebZIP33* \| 0.205079418 \| 0.66827438 \| 0.306879067 \| \| *PhebZIP100-PhebZIP21* \| 0.325698913 \| 0.781534114 \| 0.416743054 \| \| *PhebZIP101-PhebZIP108* \| 0.343029959 \| 1.167798771 \| 0.29374064 \| \| *PhebZIP103-PhebZIP101* \| 0.401943589 \| 1.088626304 \| 0.369220905 \| \| *PhebZIP104-PhebZIP8* \| 0.033236207 \| 0.053714223 \| 0.618759901 \| \| *PhebZIP104-PhebZIP36* \| 0.196218744 \| 1.41294065 \| 0.138872602 \| \| *PhebZIP106-PhebZIP22* \| 0.336079301 \| 0.479968676 \| 0.700210904 \| \| *PhebZIP106-PhebZIP131* \| 0.052321939 \| 0.105627855 \| 0.495342247 \| \| *PhebZIP106-PhebZIP40* \| 0.29284011 \| 0.482333416 \| 0.607132122 \| \| *PhebZIP107-PhebZIP4* \| 0.06405375 \| 0.15309018 \| 0.418405346 \| \| *PhebZIP107-PhebZIP116* \| 0.319841796 \| 0.881098908 \| 0.363003283 \| \| *PhebZIP107-PhebZIP10* \| 0.443035901 \| 0.842215696 \| 0.526036149 \| \| *PhebZIP107-PhebZIP23* \| 0.205834036 \| 0.594595345 \| 0.346174987 \| \| *PhebZIP108-PhebZIP127* \| 0.028515463 \| 0.163127534 \| 0.174804721 \| \| *PhebZIP109-PhebZIP26* \| 0.289261977 \| 1.093472861 \| 0.264535122 \| \| *PhebZIP110-PhebZIP128* \| 0.050381394 \| 0.121631244 \| 0.414214248 \| \| *PhebZIP110-PhebZIP39* \| 0.26965166 \| 1.05892131 \| 0.254647496 \| \| *PhebZIP111-PhebZIP126* \| 0.039353289 \| 0.128273861 \| 0.306791177 \| \| *PhebZIP112-PhebZIP47* \| 0.036151576 \| 0.152844683 \| 0.23652492 \| \| *PhebZIP113-PhebZIP9* \| 0.043927447 \| 0.098168084 \| 0.447471776 \| \| *PhebZIP114-PhebZIP5* \| 0.028904731 \| 0.13118111 \| 0.220342176 \| \| *PhebZIP114-PhebZIP144* \| 0.15453963 \| 0.62353297 \| 0.247845162 \| \| *PhebZIP115-PhebZIP7* \| 0.186429368 \| 0.226099139 \| 0.824547008 \| \| *PhebZIP115-PhebZIP145* \| 0.449296748 \| 0.577269381 \| 0.778313839 \| \| *PhebZIP116-PhebZIP4* \| 0.305174841 \| 0.83193236 \| 0.366826506 \| \| *PhebZIP116-PhebZIP10* \| 0.100090095 \| 0.13900589 \| 0.720042112 \| \| *PhebZIP117-PhebZIP6* \| 0.068434972 \| 0.160352463 \| 0.426778426 \| \| *PhebZIP117-PhebZIP148* \| 0.210942033 \| 0.633361313 \| 0.333051654 \| \| *PhebZIP118-PhebZIP138* \| 0.253260024 \| 0.883616144 \| 0.286617697 \| \| *PhebZIP118-PhebZIP151* \| 0.271006053 \| 0.90586784 \| 0.299167318 \| \| *PhebZIP118-PhebZIP149* \| 0.065477628 \| 0.129086911 \| 0.507236772 \| \| *PhebZIP119-PhebZIP147* \| 0.32407538 \| 0.737049952 \| 0.439692559 \| \| *PhebZIP119-PhebZIP11* \| 0.051011157 \| 0.198960852 \| 0.256387911 \| \| *PhebZIP121-PhebZIP136* \| 0.134897923 \| 0.490156018 \| 0.275214254 \| \| *PhebZIP121-PhebZIP13* \| 0.035669144 \| 0.112460893 \| 0.317169315 \| \| *PhebZIP123-PhebZIP139* \| 0.039794479 \| 0.705238706 \| 0.056426964 \| \| *PhebZIP124-PhebZIP12* \| 0.094650214 \| 0.210008101 \| 0.450697919 \| \| *PhebZIP128-PhebZIP39* \| 0.259991956 \| 1.164796869 \| 0.22320798 \| \| *PhebZIP128-PhebZIP26* \| 0.279207376 \| 1.34134843 \| 0.20815425 \| \| *PhebZIP129-PhebZIP27* \| 0.327212735 \| 0.733647546 \| 0.446008082 \| \| *PhebZIP131-PhebZIP22* \| 0.30858045 \| 0.579651354 \| 0.532355265 \| \| *PhebZIP131-PhebZIP40* \| 0.278649526 \| 0.524355513 \| 0.531413361 \| \| *PhebZIP133-PhebZIP18* \| 0.211106866 \| 0.493392895 \| 0.427867666 \| \| *PhebZIP135-PhebZIP14* \| 0.276885576 \| 0.653239618 \| 0.423865254 \| \| *PhebZIP136-PhebZIP13* \| 0.131984145 \| 0.491126359 \| 0.268737652 \| \| *PhebZIP138-PhebZIP151* \| 0.096687459 \| 0.148425047 \| 0.651422795 \| \| *PhebZIP138-PhebZIP149* \| 0.307028876 \| 0.820777394 \| 0.374070824 \| \| *PhebZIP142-PhebZIP16* \| 0.208029065 \| 0.820014434 \| 0.253689516 \| \| *PhebZIP144-PhebZIP5* \| 0.141469868 \| 0.592071064 \| 0.238940689 \| \| *PhebZIP147-PhebZIP11* \| 0.3325913 \| 0.96085488 \| 0.346141032 \| \| *PhebZIP148-PhebZIP6* \| 0.20676295 \| 0.663079803 \| 0.31182212 \| \| *PhebZIP149-PhebZIP151* \| 0.263548415 \| 0.89357714 \| 0.294936389 \|  \| The statistical data of the *Phe-Phe* \| \| \| \|  \|  \| \| --- \| --- \| --- \| --- \| --- \| --- \| \| **Ks value** \| numbers \| frequency \| **Ka/Ks** \| numbers \| frequency \| \| 0-0.1 \| 8 \| 0.06061 \| 0-0.1 \| 3 \| 0.02273 \| \| 0.1-0.2 \| 41 \| 0.31061 \| 0.1-0.2 \| 15 \| 0.11364 \| \| 0.2-0.3 \| 4 \| 0.03030 \| 0.2-0.3 \| 41 \| 0.31061 \| \| 0.3-0.4 \| 1 \| 0.00758 \| 0.3-0.4 \| 26 \| 0.19697 \| \| 0.4-0.5 \| 11 \| 0.08333 \| 0.4-0.5 \| 25 \| 0.18939 \| \| 0.5-0.6 \| 10 \| 0.07576 \| 0.5-0.6 \| 11 \| 0.08333 \| \| 0.6-0.7 \| 14 \| 0.10606 \| 0.6-0.7 \| 7 \| 0.05303 \| \| 0.7-0.8 \| 15 \| 0.11364 \| 0.7-0.8 \| 3 \| 0.02273 \| \| 0.8-0.9 \| 12 \| 0.09091 \| 0.8-0.9 \| 1 \| 0.00758 \| \| 0.9-1.0 \| 4 \| 0.03030 \| 0.9-1.0 \| 0 \| 0.00000 \| \| 1.0-1.1 \| 3 \| 0.02273 \|  \|  \|  \| \| 1.1-1.2 \| 4 \| 0.03030 \|  \|  \|  \| \| 1.2-1.3 \| 0 \| 0.00000 \|  \|  \|  \| \| 1.3-1.4 \| 1 \| 0.00758 \|  \|  \|  \| \| 1.4-1.5 \| 2 \| 0.01515 \|  \|  \|  \| \| 1.5-1.6 \| 0 \| 0.00000 \|  \|  \|  \| \| 1.6-1.7 \| 2 \| 0.01515 \|  \|  \|  \| \| 1.7-1.8 \| 0 \| 0.00000 \|  \|  \|  \| \| 1.8-1.9 \| 0 \| 0.00000 \|  \|  \|  \| \| 1.9-2.0 \| 0 \| 0.00000 \|  \|  \|  \| |
| --- | --- | --- | --- | --- | --- | --- | --- | --- | --- | --- | --- | --- | --- | --- | --- | --- | --- | --- | --- | --- | --- | --- | --- | --- | --- | --- | --- | --- | --- | --- | --- | --- | --- | --- | --- | --- | --- | --- | --- | --- | --- | --- | --- | --- | --- | --- | --- | --- | --- | --- | --- | --- | --- | --- | --- | --- | --- | --- | --- | --- | --- | --- | --- | --- | --- | --- | --- | --- | --- | --- | --- | --- | --- | --- | --- | --- | --- | --- | --- | --- | --- | --- | --- | --- | --- | --- | --- | --- | --- | --- | --- | --- | --- | --- | --- | --- | --- | --- | --- | --- | --- | --- | --- | --- | --- | --- | --- | --- | --- | --- | --- | --- | --- | --- | --- | --- | --- | --- | --- | --- | --- | --- | --- | --- | --- | --- | --- | --- | --- | --- | --- | --- | --- | --- | --- | --- | --- | --- | --- | --- | --- | --- | --- | --- | --- | --- | --- | --- | --- | --- | --- | --- | --- | --- | --- | --- | --- | --- | --- | --- | --- | --- | --- | --- | --- | --- | --- | --- | --- | --- | --- | --- | --- | --- | --- | --- | --- | --- | --- | --- | --- | --- | --- | --- | --- | --- | --- | --- | --- | --- | --- | --- | --- | --- | --- | --- | --- | --- | --- | --- | --- | --- | --- | --- | --- | --- | --- | --- | --- | --- | --- | --- | --- | --- | --- | --- | --- | --- | --- | --- | --- | --- | --- | --- | --- | --- | --- | --- | --- | --- | --- | --- | --- | --- | --- | --- | --- | --- | --- | --- | --- | --- | --- | --- | --- | --- | --- | --- | --- | --- | --- | --- | --- | --- | --- | --- | --- | --- | --- | --- | --- | --- | --- | --- | --- | --- | --- | --- | --- | --- | --- | --- | --- | --- | --- | --- | --- | --- | --- | --- | --- | --- | --- | --- | --- | --- | --- | --- | --- | --- | --- | --- | --- | --- | --- | --- | --- | --- | --- | --- | --- | --- | --- | --- | --- | --- | --- | --- | --- | --- | --- | --- | --- | --- | --- | --- | --- | --- | --- | --- | --- | --- | --- | --- | --- | --- | --- | --- | --- | --- | --- | --- | --- | --- | --- | --- | --- | --- | --- | --- | --- | --- | --- | --- | --- | --- | --- | --- | --- | --- | --- | --- | --- | --- | --- | --- | --- | --- | --- | --- | --- | --- | --- | --- | --- | --- | --- | --- | --- | --- | --- | --- | --- | --- | --- | --- | --- | --- | --- | --- | --- | --- | --- | --- | --- | --- | --- | --- | --- | --- | --- | --- | --- | --- | --- | --- | --- | --- | --- | --- | --- | --- | --- | --- | --- | --- | --- | --- | --- | --- | --- | --- | --- | --- | --- | --- | --- | --- | --- | --- | --- | --- | --- | --- | --- | --- | --- | --- | --- | --- | --- | --- | --- | --- | --- | --- | --- | --- | --- | --- | --- | --- | --- | --- | --- | --- | --- | --- | --- | --- | --- | --- | --- | --- | --- | --- | --- | --- | --- | --- | --- | --- | --- | --- | --- | --- | --- | --- | --- | --- | --- | --- | --- | --- | --- | --- | --- | --- | --- | --- | --- | --- | --- | --- | --- | --- | --- | --- | --- | --- | --- | --- | --- | --- | --- | --- | --- | --- | --- | --- | --- | --- | --- | --- | --- | --- | --- | --- | --- | --- | --- | --- | --- | --- | --- | --- | --- | --- | --- | --- | --- | --- | --- | --- | --- | --- | --- | --- | --- | --- | --- | --- | --- | --- | --- | --- | --- | --- | --- | --- | --- | --- | --- | --- | --- | --- | --- | --- | --- | --- | --- | --- | --- | --- | --- | --- | --- | --- | --- | --- | --- | --- | --- | --- | --- | --- | --- | --- | --- | --- | --- | --- | --- | --- | --- | --- | --- | --- | --- | --- | --- | --- | --- | --- | --- | --- | --- | --- | --- | --- | --- | --- | --- | --- | --- | --- | --- | --- | --- | --- | --- | --- | --- | --- | --- | --- | --- | --- | --- | --- | --- | --- | --- | --- | --- | --- | --- | --- | --- | --- | --- | --- | --- | --- | --- | --- | --- | --- | --- | --- | --- | --- | --- | --- | --- | --- | --- | --- | --- | --- | --- | --- | --- | --- | --- | --- | --- | --- | --- | --- | --- | --- | --- | --- | --- | --- | --- | --- | --- | --- | --- | --- | --- | --- |
