# Supplementary material for: Genome-Wide Identification and Expression Analyses of the bZIP Transcription Factor Genes in moso bamboo (Phyllostachys edulis)
Source: Int J Mol Sci. 2019 May 5;20(9):2203. doi: 10.3390/ijms20092203 (PMC6539497; doi:10.3390/ijms20092203)
Supplement: Supplementary file 1 [file ijms-20-02203-s001.zip › Supplementary/Table S5.docx]

| **Table S5.** Oligonucleotide primers used in qRT-PCR assays for selected 18 *PhebZIP* genes | | |
| --- | --- | --- |
| Gene | | Primer sequence (5′→3′) |
| *PhebZIP21* | Forwards | TGAGGAACCGGGTGTCGG |
|  | Reverse | TCTCCAGGTCCTTGACCTTCG |
| *PhebZIP23* | Forwards | ATGGTGCCTGGGGTCGTC |
|  | Reverse | CCTTGCCCAACCCATTCAG |
| *PhebZIP25* | Forwards | GGCAAGGAAGAGGGCATACG |
|  | Reverse | TTGATGAGGTTCGTCGCAGTG |
| *PhebZIP26* | Forwards | CGAGTTGCAGGTCGAAC |
|  | Reverse | AGCGAGTTCTGAACTTGTCGAG |
| *PhebZIP36* | Forwards | AGAGCCGTTTCTTGATCGTGC |
|  | Reverse | GGCTCATCTGCTTCTGCTTCC |
| *PhebZIP47* | Forwards | ACGAGCGCAAGCGCAAGA |
|  | Reverse | CGACTCCCTGTTCGACAGCAT |
| *PhebZIP59* | Forwards | ATGTCGTCGTCGTCGCTGTC |
|  | Reverse | TCCAGGTGCTGCTGCTTCC |
| *PhebZIP68* | Forwards | GAAGCCATGCCATCCAC |
|  | Reverse | CGTGCATCTGCTTCAGCTTC |
| *PhebZIP72* | Forwards | GTTGCGAAGTGCGGCTGAC |
|  | Reverse | CACTGGTGCCTTGTGGGAAAT |
| *PhebZIP77* | Forwards | CCAGGCGGGTCAAGAGGA |
|  | Reverse | CGGTCAGATTCACGGTGTTGTT |
| *PhebZIP88* | Forwards | TCCGCAACGATGCCCTCA |
|  | Reverse | CCTCTTCAATTCCTGCTCACCC |
| *PhebZIP92* | Forwards | GAGCTGACGGCGCAGGTGG |
|  | Reverse | AAGGGACTGGAGGCGGTGG |
| *PhebZIP97* | Forwards | CAGATGCCAGCCCAAGAACA |
|  | Reverse | TGGAGTCGGAAGCAACAACG |
| *PhebZIP105* | Forwards | TGGGAGGCTCAAGTGGTTCA |
|  | Reverse | CCATTGGGTTTGTGCTTTGC |
| *PhebZIP117* | Forwards | CTTGAGCGTAAGGTTCAGGTGC |
|  | Reverse | GCAAGCCCAGCATAGTCCC |
| *PhebZIP126* | Forwards | AGAACGCCCACGTCGTCA |
|  | Reverse | CGGCACCGTTGTTGCTCAT |
| *PhebZIP142* | Forwards | CTGAGTCGCCGTTCGTGG |
|  | Reverse | GCATTCACATCCTCCTGTCCTG |
| *PhebZIP144* | Forwards | TGCTCTGAATGGCACCCTAAA |
|  | Reverse | TGAAACATCTGCTGGTTCCGTC |
| *TIP41* | Forwards | AAAATCATTGTAGGCCATTGTCG |
|  | Reverse | ACTAAATTAAGCCAGCGGGAGTG |
