# Supplementary figures and images for: Genome-Wide Identification and Expression Analyses of the bZIP Transcription Factor Genes in moso bamboo (Phyllostachys edulis)
Source: Int J Mol Sci. 2019 May 5;20(9):2203. doi: 10.3390/ijms20092203 (PMC6539497; doi:10.3390/ijms20092203)

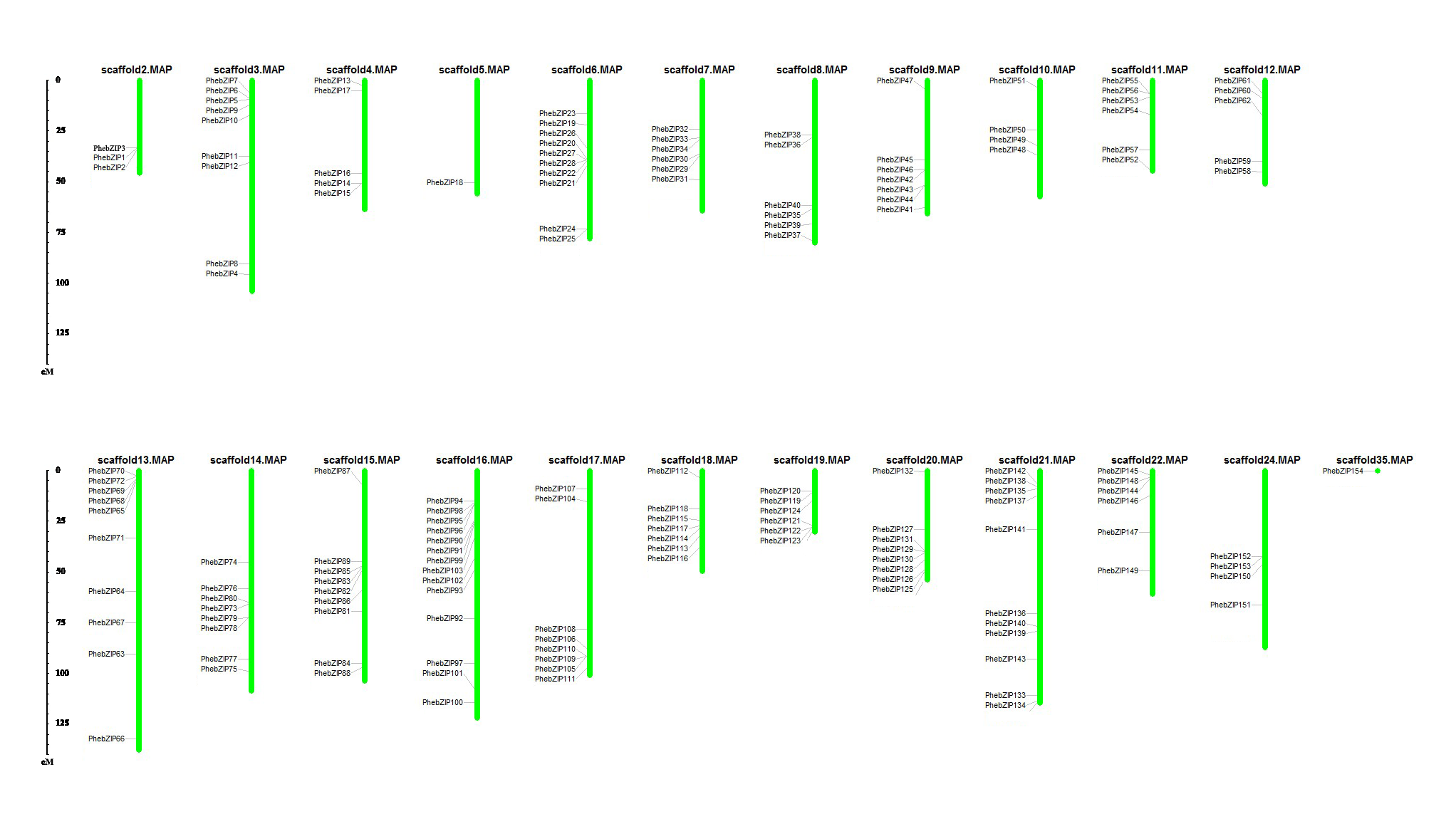

Supplement: Supplementary file 1 [file ijms-20-02203-s001.zip › Supplementary/Figure S1.tif]
